# Guard‐cell expression of abscisic acid receptors for engineering water‐use‐efficient plants without trade‐offs in growth

**DOI:** 10.1111/nph.70404

**Published:** 2025-07-24

**Authors:** Jinghui Liu, Rudi Schäufele, Alexander Christmann, Mutez Ahmed, Zhenyu Yang

**Affiliations:** ^1^ Chair of Botany, School of Life Sciences Technical University of Munich Emil‐Ramann‐Str. 4 Freising 85354 Germany; ^2^ Chair of Crop Physiology, School of Life Sciences Technical University of Munich Alte Akademie 12 Freising 85354 Germany; ^3^ Chair of Root‐Soil Interaction, School of Life Sciences Technical University of Munich Emil‐Ramann‐Str. 4 Freising 85354 Germany

**Keywords:** ABA receptor, abscisic acid, growth trade‐offs, guard cell, leaf water potential, stomata, transpiration, water use efficiency

## Abstract

- Abscisic acid (ABA) induces stomatal closure, reducing transpirational water loss, a critical adaptation for agriculture under drought. However, ABA is often viewed cautiously as stomatal closure limits CO_2_ uptake for photosynthesis and growth. We have demonstrated that ectopic expression of selected ABA receptors in Arabidopsis reduces transpiration without growth penalty, thus achieving high water use efficiency (WUE). The tissues and mechanisms underlying this trait remain unclear, though prior evidence suggests a significant contribution from shoot ABA responsiveness. We hypothesize that guard‐cell ABA signaling contributes to the trait of high WUE without growth penalty.
- We generated lines overexpressing 14 individual ABA receptors under the guard cell–specific promoter and examined leaf growth, transpiration, water potential, and net carbon assimilation rate (*A*_N_).
- We found that guard‐cell overexpression of specific ABA receptors reduced transpiration and *A*_N_ compared to the wild‐type under well‐watered conditions, but leaf growth was not adversely affected. The mechanism involved receptor‐mediated reduction in transpiration and resultant improved leaf water potential for efficient intermediate‐aged leaf growth. Under drought, these lines showed enhanced WUE without compromising biomass accumulation.
- Our findings highlight activation of ABA signaling in guard cells as a promising strategy for developing water‐saving crops without growth trade‐offs.

## 摘要

- 脱落酸(ABA) 能诱导气孔关闭、减少蒸腾失水，是农业抗旱的关键调节因子。然而，由于气孔关闭也会限制光合作用所需的 CO_2_ 吸收，ABA 常被谨慎使用。我们之前的研究已经证明，在拟南芥中 过度表达特定 ABA 受体可在不影响生长的情况下降低蒸腾，从而实现高水分利用效率(WUE)。赋予这一表型的相关组织与机制尚未厘清，但已有证据显示地上部 ABA 的作用贡献显著。本研究假设:保卫细胞中的 ABA 信号转导是在不影响植物生长的前提下提高 WUE 的关键。
- 我们构建了保卫细胞特异启动子驱动下过表达14个单一 ABA 受体的转基因系，并测定了叶片生长、蒸腾速率、净碳同化速率(*A*_N_)及叶水势等指标。
- 结果表明，在充足供水条件下，特定受体的保卫细胞过表达显著降低了蒸腾和*A*_N_，但叶片生长未受抑制。可能的机制为:受体介导的蒸腾降低提升了叶水势，进而促进中龄叶的高效生长。在干旱处理中，这些转基因系表现出更高的 WUE，同时生物量积累未受影响。
- 综上，激活保卫细胞 ABA 信号为培育节水且无生长抑制的作物提供了有前景的分子策略。

## Zusammenfassung

- Abscisinsäure (ABA) induziert das Schließen der Stomata und reduziert so den Wasserverlust durch Transpiration – ein für die Landwirtschaft bei Trockenheit entscheidender Anpassungsmechanismus. Da der Stomataschluß jedoch auch die CO_2_‐Aufnahme für die Photosynthese limitiert, wird der Einfluß der ABA jedoch meist als kritisch angesehen. Wir hatten gezeigt, dass die ektopische Expression ausgewählter ABA‐Rezeptoren in Arabidopsis die Transpiration ohne Wachstumseinbußen verringert und damit eine hohe Wassernutzungseffizienz (WUE) erzielt wird. Welche Gewebe und Mechanismen für diese Beobachtung ausschlaggebend sind, ist noch ungeklärt; frühere Daten deuten jedoch auf einen starken Beitrag der ABA‐Antwort des Sprosses hin. Wir stellen die Hypothese auf, dass die ABA‐Signaltransduktion in Schließzellen entscheidend zu einer hohen WUE ohne Wachstumseinbußen beiträgt.
- Wir haben Linien erzeugt, die vierzehn ABA‐Rezeptoren von Arabidopsis jeweils einzeln unter einem schließzellspezifischen Promotor überexprimieren, und haben Blattwachstum, Transpiration, Netto‐Kohlenstoff‐Assimilationsrate (*A*_N_) sowie Blattwasserpotential bestimmt.
- Die Schließzellen‐Überexpression bestimmter ABA‐Rezeptoren senkte bei guter Wasserversorgung Transpiration und *A*_N_ gegenüber dem Wildtyp, ohne das Blattwachstum negativ zu beeinflussen. An diesem Effekt beteiligt sind eine rezeptorvermittelte Transpirationsreduktion und ein daraus resultierendes verbessertes Blattwasserpotential, das ein effizientes Wachstum von Blättern mittleren Alters ermöglichte. Unter Trockenstress zeigten die entsprechenden Linien eine erhöhte WUE, ohne dass die Biomasseakkumulation beeinträchtigt wurde.
- Unsere Ergebnisse heben die Aktivierung der ABA‐Signaltransduktion in Schließzellen als vielversprechende Strategie hervor, um Nutzpflanzen Kulturpflanzen ohne Wachstumsbeeinträchtigungen zu entwickeln.

## Introduction

Freshwater is vital to crop production. However, current and future freshwater availability is challenged by climate change and groundwater depletion, threatening global food security (FAO, 2021). Therefore, crops fitting into the future scenario of water deficit and possessing high water use efficiency (WUE) are urgently needed. WUE is a multifaceted concept defined as the ratio of yield to water input at the agricultural scale, biomass produced to consumed water at the whole‐plant scale, or *A*_N_ to transpiration rate (*E*) or stomatal conductance (*g*_s_) at the leaf level (Hatfield & Dold, 2019). Despite significant efforts invested in developing high‐WUE crops, current research and practice indicates that breeding crops with high WUE often leads to trade‐offs in growth and yield under non‐drought conditions (Blum, 2005). These observations raise questions over whether it is possible to enhance WUE without compromising growth.

Plant water loss primarily occurs through stomatal pores, which are formed by paired guard cells (Brodribb & McAdam, 2011). As water moves along the water potential gradient from regions of high to low potential and due to the nearly saturated water vapor in the substomatal cavity (the inner cavity of the leaf immediately adjacent to the stomata) (Wong *et al*., 2022), the loss of water from the leaf is mostly unavoidable according to Fick's law of diffusion. When leaf water homeostasis is suboptimal, higher plants promote closing of stomatal apertures to mitigate leaf water loss via mechanisms that lead to loss of turgor in guard cells, triggered by biochemical modulations primarily by the phytohormone Abscisic acid (ABA) signaling pathways and, yet largely unknown, hydraulic signal transduction (Christmann *et al*., 2013).

Under drought, ABA levels increase and induce physiological adjustments that involve the homeostasis of the water status by reducing stomatal aperture and, thus, transpiration (Hsu *et al*., 2021). The ABA signal is perceived by a holo‐receptor complex formed by the ABA‐binding Regulatory Component of ABA Receptor (RCAR)/Pyrabactin Resistance 1 (PYR1)/PYR1‐Like together with the catalytically active ABA coreceptor, type 2C protein phosphatases (PP2Cs) (Ma *et al*., 2009; Melcher *et al*., 2009; Park *et al*., 2009; Santiago *et al*., 2009). The phosphatase activity of the ABA coreceptors inhibits downstream‐acting protein kinases sucrose non‐fermenting 1–related subfamily 2 kinases (SnRK2s) (Melcher *et al*., 2009; Santiago *et al*., 2009). However, the catalytic activity of PP2Cs becomes blocked by the formation of the holo‐receptor complex. SnRK2s are activated by phosphorylation either by their own activity exemplified by OST1/SnRK2.6/SnRK2E (Umezawa *et al*., 2009; Ruschhaupt *et al*., 2019) or via upstream protein kinases such as the rapidly accelerated fibrosarcoma (RAF)‐like protein kinases (Lin *et al*., 2020; Soma *et al*., 2020; Takahashi *et al*., 2020). In guard cells, active SnRK2.6 subsequently activates the guard‐cell slow (S)‐type anion channel (Geiger *et al*., 2009; Lee *et al*., 2009), inhibits the inwardly rectifying (transporting) K^+^ channel (KAT1) (Sato *et al*., 2009), and activates plasma membrane aquaporins (PIP2.1) (Grondin *et al*., 2015) to induce stomatal closure. This action of ABA reduces leaf water loss, positioning ABA, and the ABA signaling pathway as promising targets for alleviating the impact of water deficit and for saving water under non‐drought conditions (Cao *et al*., 2017; Vaidya *et al*., 2019; Yang *et al*., 2019; Roeder *et al*., 2023).

Activation of ABA signaling imposes a detrimental impact on leaf growth over time under well‐watered conditions (Milborrow, 1974; Yang *et al*., 2019). These observations can be explained by a prevailing opinion that reducing stomatal aperture and, thereby, *g*_s_ by ABA to limit water diffusion also hinders the influx of CO_2_, leading to lowered internal CO_2_ availability for photosynthesis (Condon, 2020). As a result, cellular carbon deposition is reduced, ultimately exerting an adverse effect on leaf growth (Hilty *et al*., 2021). In addition to carbon‐dependent growth, leaf growth also relies on expansive cell growth, which involves cell wall loosening and volumetric cell enlargement through cell water influx (Kalve *et al*., 2014). The latter is closely associated with plant hydraulic status and the difference in water potential between the growing cells and the surrounding apoplasts (Tardieu *et al*., 2010). ABA affects water homeostasis in plants. It is intuitive that leaf transpiration reduces leaf water potential, and the antitranspirational effect of ABA likely mitigates such a reduction. In addition, ABA also regulates leaf and root hydraulic conductivity (Parent *et al*., 2009; Pantin *et al*., 2013; Shahzad *et al*., 2024). However, the extent to which ABA‐mediated hydraulic regulation and leaf water status impact leaf growth has not yet been fully understood.

Our previous research revealed that activating specific ABA signaling via ectopic expression of each of 14 individual RCARs driven by the cauliflower virus promoter (*p35S* promoter) gave rise to distinct leaf growth phenotypes (Yang *et al*., 2016), although a majority of the possible 126 RCAR‐PP2C pairs can functionally activate ABA signaling over a wide range of ABA levels (Tischer *et al*., 2017). Among these receptor overexpressors, p35S:RCAR6 and p35S:RCAR10 lines achieved reduced transpiration and high WUE but without growth penalty (Yang *et al*., 2016, 2019). Similar traits have also been observed in crop species through ABA receptor manipulation in wheat and maize (Mega *et al*., 2019; Mao *et al*., 2022). It is clear that the p35S:RCAR6 line reduced *g*_s_ to restrict leaf transpiration and, meanwhile, to limit CO_2_ uptake (Yang *et al*., 2016), but the question remains as to how plants can maintain leaf growth at lowered internal CO_2_ concentration. Tissue specificity of ABA receptor action might explain, to some extent, the differences between ABA receptors on growth. The high WUE without growth penalty of receptor overexpressors is mediated by its expression in the shoot, as shown by the grafting analysis (Yang *et al*., 2016). These findings suggest that there are potential mechanisms compensating for the lowered internal CO_2_ for maintaining leaf growth, which may be implicated in CO_2_ concentration mechanisms or mechanisms related to controlling leaf water status. To test these possibilities in this study, we first addressed to what extent a selective expression of ABA receptors in guard cells can recapitulate the phenotypes observed previously by whole‐plant expression of ABA receptors (Yang *et al*., 2016, 2019) and next examined the physiological mechanisms of leaf growth regulated by the receptor‐mediated ABA signaling in guard cells. We generated transgenic Arabidopsis lines overexpressing each of the 14 ABA receptors in guard cells, driven by the guard cell–specific promoter, *pGC1* (Yang *et al*., 2008). Overexpressing several subfamily II ABA receptors in guard cells combined reduced leaf transpiration with maintained leaf growth compared to the wild‐type (wt) accession under well‐watered conditions. Detailed analyses using pGC1:RCAR6 lines revealed that the potential physiological mechanism involved RCAR6‐mediated reduction in leaf transpiration and resultant improved leaf water potential for efficient leaf growth.

## Materials and Methods

### Chemicals and plant materials

Chemicals with the highest purity were obtained from Sigma‐Aldrich (www.sigmaaldrich.com), ROTH (www.carlroth.com), and J.T. Baker (http://www.avantormaterials.com). (S)‐ABA was purchased from CHEMOS (www.chemos.de). The Arabidopsis (*Arabidopsis thaliana* (L.) *Heynh*) accession Columbia (Col‐0) was received from the Nottingham Arabidopsis Stock Center. Transgenic lines ectopically expressing RCAR6 were established by transforming an expression cassette for *RCAR6* driven by the *p35S* promoter into the wt, as described by Yang *et al*. (2016).

Constructs enabling guard‐cell constitutive expression of 14 ABA receptors were generated using the multi‐kingdom Golden Gate assembly platform (Chiasson *et al*., 2019). Briefly, 1716 bp upstream of *ATG* of At1g22690 (*GC1*) from the wt genomic DNA and ABA receptors from cDNA were amplified using primers listed in Supporting Information Table S1. The resultant fragments were cloned into the *Level I (LI)* vector (*pSEVA641*) using BpiI restriction enzyme to generate *LI_A‐B_pGC1* and *LI_C‐D_RCAR1‐14* (“A‐B” and “C‐D” indicate vector entry sequence in the *LII backbone*). Expression constructs (*LII_F2‐3_pGC1:GFP‐RCAR1‐14*) were assembled in *LIIc_F2‐3* backbone using BsaI restriction enzyme together with *LI_B‐C_GFP*, *LI_D‐E_dummy element (dy)*, *LI_E‐F_Nos terminator*, and *LI_F‐G_dy*. The final assembly was achieved by including the Basta resistance gene expression cassette and LIII dummy elements using BpiI again. The resulting constructs, *LIII_βfin_LII_1–2_Basta_LII_2–3_pGC1:GFP‐RCAR1‐14_LII_3‐4_dy_LII_4‐5_dy* (‘1–2’, ‘2–3’, etc., indicate their sequence on the LIII backbone), were transformed into wt plants to generate transformants specifically expressing ABA receptors in guard cells. All transgenic lines were selected for homozygosity as carried out for RCAR1 (Ma *et al*., 2009). Homozygous lines of the T3 generation were used for analyses in this study.

### Plant growth conditions

Arabidopsis seeds were sterilized by a two‐step washing process using 80% ethanol followed by 3% sodium hypochlorite. Sterilized seeds were sown on 1/2‐strength Murashige & Skoog agar medium and stratified at 4°C for 2 d. The seeds were next incubated for 7 d at 60 μmol m^−2^ s^−1^ photosynthetic active radiation (PAR) and 22°C. Established seedlings with comparable leaf size were transferred to 0.2 l pots filled with soil (Classic Profi Substrate, Einheitserde Werkverband, Sinntal‐Altengronau, Germany). Soil water content (SWC) was controlled according to the experimental design. All plants were grown in plant growth cabinets (Conviron E15, Winnipeg, MB, Canada) or at the Technical University of Munich EcoSystem Analyzer (TUMmesa; Jákli *et al*., 2021) under a short‐day light regime with 8 h light : 16 h dark at 150 μmol m^−2^ s^−1^ PAR, and temperature and relative humidity were 22°C, 50% during daytime and 17°C, 60% at night.

### Protoplast analyses

Preparation and analysis of protoplasts was performed as described (Tischer *et al*., 2017). Leaves of 3‐wk‐old long‐day grown (16 h light : 8 h dark) wt plants were cut off and incubated in an enzyme solution, a mixture of Onozuka R‐10 and Macerozym R‐10 (Yakult, Tokyo, Japan) for 4 h to lyse the cell wall. Careful washing steps were taken, and the final concentration of living protoplasts was adjusted to 10^5^ protoplasts per ml. Next, the protoplasts were transfected with plasmid DNA, including 3 μg *pRD29B:LUC* construct as an ABA‐responsive reporter, 3 μg *p35S:GUS* expression cassette as an internal control for normalization, and 3 μg *p35S:GFP, p35S:RCAR6, p35S:GFP‐RCAR6*, *or p35S:RCAR6‐GFP* as effector DNA. An empty vector with the same expression cassette served as a control for comparison. The protoplast suspension was supplemented without or with 3 μmA ABA according to the experimental design. The transfected protoplasts were then incubated at 22°C for 18 h. The expression of luciferase (LUC) and β‐glucuronidase (GUS) was determined by using a HIDEX plate luminometer (Turku, Finland) and a Synergy 2 plate reader (Bio‐Tec, Winooski, VT, USA), respectively.

### Confocal microscopy

The FV3000 confocal laser scanning microscope (Olympus Europe, Hamburg, Germany) was used to perform confocal microscopy. To achieve high‐resolution images, we employed a 60× water immersion objective (NA of 1.2) and a high sensitivity detector and set the scan speed to 4.0 μs per pixel (1024 × 1024 pixels image), a line average of 2, and a digital zoom of 1. Excitation emission was achieved by using a 488 nm 500 mW diode laser at 0.9% intensity. Emission of green fluorescent protein (GFP) was detected at 500–540 nm. Chlorophyll autofluorescence, if applicable, was detected at 630–670 nm. All images were scaled in imagej/fiji (Schindelin *et al*., 2012) and composites of GFP and bright‐field images were created using the channel function in the same software.

### Isolation of the guard cells

Guard cells are isolated as described by Bauer *et al*. (2013) with some modifications. Briefly, fully developed leaves of 55‐d‐old plants were harvested and the midvein removed. The harvested leaves were blended five times in an ice‐cold blender (Braun MX2050). The debris was filtered and rinsed each time. A paper towel was used to remove the water of the resultant debris quickly. The debris was then loaded in an Eppendorf tube and frozen in liquid nitrogen.

### RNA extraction, cDNA synthesis, and quantitative real‐time PCR

Total RNA of the guard cells was extracted using the innuPREP Plant RNA Kit (Analytic Jena, https://www.analytik‐jena.de/). One microgram of total RNA was then used for first‐strand cDNA synthesis, achieved by using the First‐Strand cDNA Synthesis Kit (Thermo Fisher, https://www.thermofisher.com/de/). The real‐time PCR was prepared using the DNA Master SYBR Green I kit (Promega, https://www.promega.de/) and performed on the LightCycler 480 instrument (Roche Diagnostics, Penzberg, Germany). The primers used are listed in Table S2.

### Imaging

Optical imaging was conducted by using a Canon camera (PowerShot G10; Canon, Tokyo, Japan). Single plants were imaged, and pictures were aligned according to their genotype.

Thermal imaging was carried out as described (Yang *et al*., 2016). The InfraTec instrument (IR‐TCM 384; InfraTec, Dresden, Germany) was employed to image plants grown in trays containing 24 pots. The position of pots was randomized, and the analysis was done in the plant growth cabinet. The leaf temperature was analyzed using irbis3 plus software (InfraTec) and expressed as the averaged surface temperature of the whole rosette.

Pulse Amplitude Modulation (PAM) imaging was performed by using a MAXI version of IMAGING PAM (Heinz Walz, Effeltrich, Germany). The operation of the PAM imaging system was according to the manufacturer's instructions. In brief, the plantlets were placed under the camera, focused, and then dark adapted for 30 min. Next, the minimal level of fluorescence (*F*_o_) was recorded, and a saturating light was triggered to obtain the maximal fluorescence level (*F*_m_) for estimating the maximum photosystem II (PS II) quantum yield. Subsequently, the actinic light was set to 150 μmol m^−2^ s^−1^ PAR. After 1 h of illumination, the transient fluorescence (*F*_t_) was determined, and a saturating light pulse was provided to determine the maximal fluorescence level (*F*_m_′) under illumination. The quantum yield of PS II under illumination was calculated as (*F*_m_′–*F*_t_)/*F*_m_′.

### Gas exchange analysis

A GFS3000 gas exchange system (Heinz Walz, Effeltrich, Germany) configured with a standard measuring head, or a custom‐built whole plant cuvette (ring chamber) was used to determine the *A*_N_ and *E* of single leaf and whole rosette, respectively. The gas exchange analyses were conducted at 150 or 900 μmol m^−2^ s^−1^ PAR at 23°C (leaf temperature). CO_2_ was set to 400 μmol mol^−1^ and leaf‐to‐air vapor pressure deficit (VPD) was controlled at 1.2 ± 0.1 kPa. The *g*_s_ was estimated based on the photosynthetic model embedded in the attached software, and intrinsic WUE (iWUE) was calculated as the ratio of *A*_N_ to *g*_s_.

*A*_N_–C_i_ curves were generated by using the whole‐rosette configuration at 900 μmol m^−2^ s^−1^ PAR with a leaf temperature of 23°C and a VPD of 1.15 ± 0.15 kPa. *A*_N_ was determined at an initial ambient CO_2_ level (C_a_) of 400 μmol mol^−1^ for 30 to 60 min to reach constant *A*_N_ and *E*. C_a_ was then decreased stepwise down to 50 μmol mol^−1^. Upon completion, C_a_ was returned to 400 μmol mol^−1^ directly to restore the original *A*_N_. Once achieved, C_a_ was increased stepwise to 1600 μmol mol^−1^. For each C_a_ except 400 μmol mol^−1^, gas exchange parameters were recorded for 5 min as soon as *A*_N_ stabilized. Leakage of CO_2_ was determined by monitoring ^13^C composition of the air at the inlet and outlet of the ring chamber with zero and 1600 μmol mol^−1^ C_a_ (with the empty ring chamber).

### Stomatal density, size, and aperture

Stomatal traits were assessed using the abaxial epidermis of the 14^th^ leaf from 25‐d‐old wt, p35S:RCAR6, and pGC1:RCAR6 plants. The selected leaf was excised, and its adaxial surface was immediately adhered to a transparent adhesive tape mounted on a microscope slide. A second piece of tape was used to peel away the abaxial epidermis, which was then transferred onto another slide for imaging. Images were captured using a SWIFT digital camera (Swift Optical Instruments, Schertz, TX, USA) connected to a Carl Zeiss Axioskop microscope (HBO 50 Axioskop; Carl Zeiss, Oberkochen, Germany). Stomatal density, size, and aperture were quantified using imagej software. Stomatal density was determined as the number of stomata mm^−2^. Stomatal size was calculated as an area of an ellipse (π × width/2 × length/2). Stomatal aperture was expressed as the ratio of stomatal pore width to length.

### Leaf area, leaf number, and leaf mass per area

The leaf area was expressed as projected rosette size (unless otherwise stated) and was quantified by comparing pixel values of the rosette with an area‐known reference using photoshop elements software (Adobe, San Jose, CA, USA). Leaf numbers were obtained by tracing and numbering the leaves according to the sequence of their emergence. To determine leaf fresh and dry mass per area, the midvein was first removed before recording the leaf area. The fresh weight was recorded immediately, and the leaf dry mass was recorded after placing the samples in the oven at a temperature of 60°C for 3 d. The ratio of leaf fresh mass to area and of leaf dry mass to area was then calculated. The single‐leaf dry biomass was determined as the dry biomass of the removed midvein and the rest of the leaf that was previously used for the dry biomass‐derived leaf mass per area (LMA).

### Leaf water potential, osmotic potential, and turgor pressure

The leaf water potential, osmotic potential, and turgor pressure were analyzed at predawn and predusk. The 24^th^, 25^th^, and 26^th^ leaves of 58‐ to 60‐d‐old plants were excised at the leaf petiole and immediately placed in a pressure chamber (Model 1002; PMS Instrument Company, Albany, OR, USA). The pressure was increased slowly until the flow of water just appeared at the cutting interface, and this equilibrium pressure was recorded as the leaf water potential. The same leaf was then frozen in liquid nitrogen and thawed at room temperature, followed by centrifuging at 18 000 ***g***. The resultant leaf sap was then measured with a vapor pressure osmometer (5100C; Wescor Inc., Logan, UT, USA) according to the manufacturer's instructions. The leaf turgor pressure was calculated as the difference between water potential and osmotic potential.

### Water deficit experiments

#### Progressive drought

The progressive drought experiment is based on the methodology outlined in Yang *et al*. (2016). Briefly, Arabidopsis plantlets were grown under well‐watered and short‐day conditions for 18 d followed by transferring to 0.2‐l pots loaded with water‐saturated soil. The soil surface was sealed to prevent evaporation, and no water was administered afterwards. The water deficit slowly developed through transpirational water loss from the plants. The experiment lasted for 9 wk until all the plant available water was consumed. The plants' rosette was photographed, and the pot weight was recorded repeatedly during the progressive drought. The rosette size is expressed as the projected leaf area. Consumed water was calculated as the difference in the pot weight compared to that at the onset of the drought. Aboveground material was harvested at the end of the drought, and biomass was determined after drying the leaf material to constant weight (3 d at 60°C). The WUE is calculated as the ratio of aboveground dry biomass to consumed water.

#### Deficit irrigation

Seven‐d‐old seedlings of wt, p35S:RCAR6, and pGC1:RCAR6 lines were transferred to 200‐ml pots filled with saturated fine soil (field capacity is *c*. 77%). To initiate the water deficit, water was not provided to plants for 7 d to reduce SWC to *c*. 20%. The soil surface was then covered to minimize evaporation, and SWC was either adjusted to 60% (well‐watered) or maintained at 20% (water deficit). This water regime lasted 35 d, during which pot weights were recorded regularly to maintain target SWC. At the end of the treatment, shoot dry biomass was determined. WUE was calculated as the ratio of shoot dry biomass to the total water consumed during the experimental period.

### ^13^Carbon isotope analysis and integrated water use efficiency

Carbon isotope composition (δ^13^C, in ‰) of whole‐shoot dry biomass was analyzed as described in Yang *et al*. (2019). ^13^C discrimination (∆^13^C, in ‰) was calculated as ∆^13^C = (δ^13^C_air_−δ^13^C_plant_)/(1 + δ^13^C_plant_/1000) (Farquhar *et al*., 1989). Integrated WUE is defined as integrated WUE = *A*_N_/*g*_s_ = 0.625 (C_a_−C_i_) = 0.625 C_a_ (1−C_i_/C_a_) (Franks *et al.,* 2013). The factor 0.625 gives the ratio of the diffusivities of CO_2_ and water vapor in air. In the Conviron growth cabinets, C_a_ was 400 mmol mol^−1^, with an approximated δ^13^C_air_ value of −9.2‰. The ‘simplified’ Farquhar model based on nonlimiting mesophyll conductance was applied to estimate C_i_/C_a_: ∆^13^C = *a* + (*b*–*a*) C_i_/C_a_, where *a* (4.4‰) denotes the fractionation of ^13^CO_2_ relative to ^12^CO_2_ during diffusion through the stomatal pores, and *b* (27.6‰) denotes the net fractionation during carboxylation reactions.

### Statistical analysis

All data were analyzed using Microsoft Excel 365 or Origin 2023. Statistical significance and linear curve fitting were achieved by one‐way ANOVA (Tukey test) and Origin 2023, respectively.

## Results

### The *pGC1* promoter provides guard cell–specific expression of abscisic acid receptor RCAR6

**Fig. 1:**
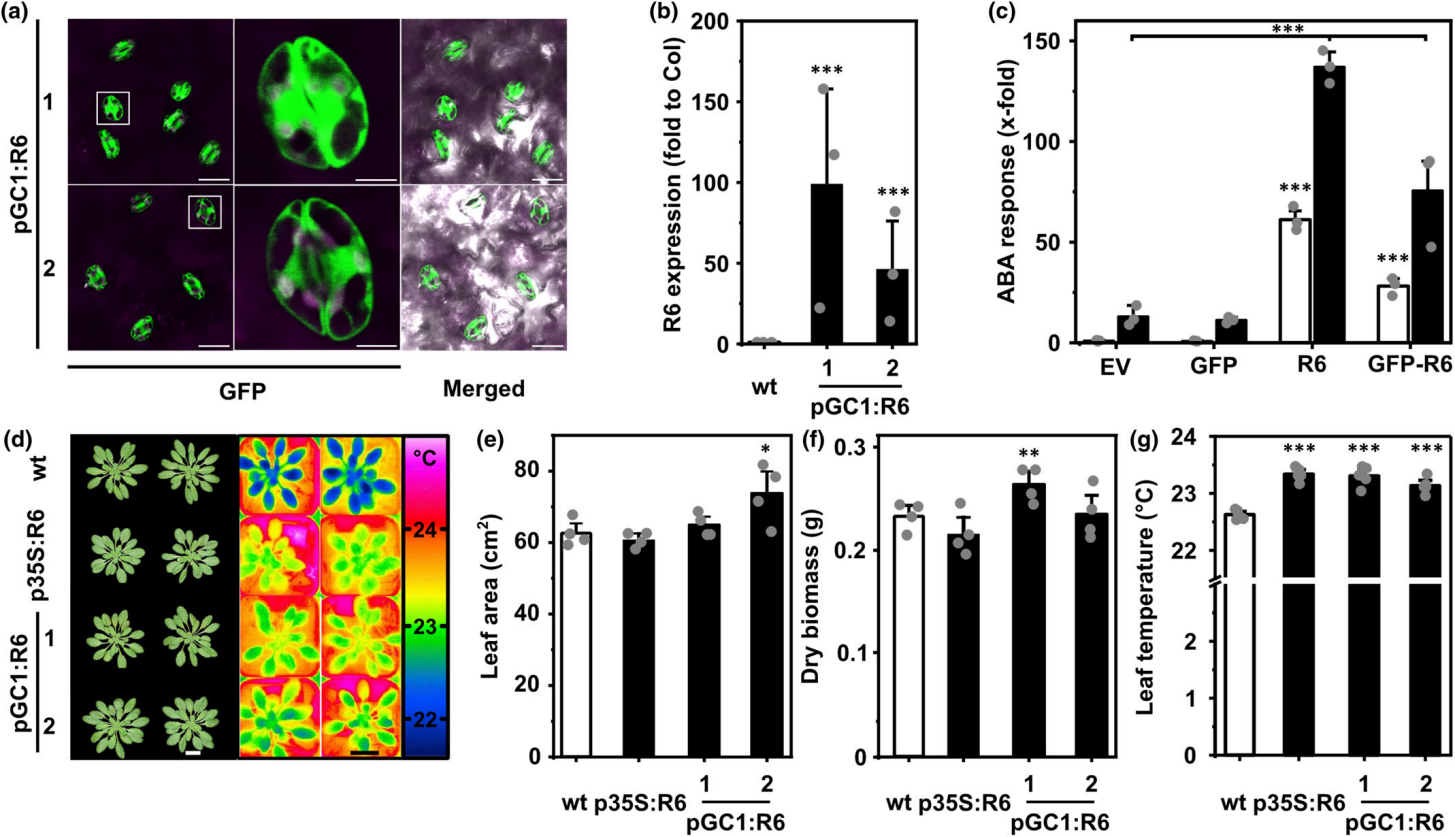
Expression of green fluorescent protein (GFP)‐tagged abscisic acid (ABA) receptor RCAR6 under the guard cell‐specific promoter *pGC1* confers reduced transpiration without trade‐offs in leaf growth. (a) Confocal analysis of abaxial leaf epidermis for GFP fluorescence from 14‐d‐old seedlings of two representative pGC1:RCAR6 (R6) lines expressing the aminoterminally tagged RCAR6. Left panel: GFP fluorescence (green) and Chl autofluorescence (magenta). Middle panel: magnified single stoma framed in white on the left. Right panel: merge of fluorescence and bright‐field image. The scale bars on the left and right panels indicate 20 μm, whereas the scale bars on the middle panels represent 5 μm. (b) *RCAR6* transcript level in guard cells of both pGC1:R6 lines compared to the wt. Guard cells were isolated, and the *RCAR6* transcript level was normalized to Arabidopsis ubiquitin 10 (AT4G05320). Three biological replicates with three technical replicates for each sample, mean ± SE. (c) The GFP‐tagged RCAR6 (R6) triggers ABA signaling in Arabidopsis protoplasts. Reporter constructs expressing an ABA‐responsive luciferase (LUC) reporter and a constitutively expressed GUS reporter were transfected into wild‐type (wt) protoplasts together with effector constructs containing expression cassettes of *p35S:GFP (GFP)*, *p35S:R6*, or *p35S:GFP‐R6*. The empty vector (EV) containing the same promoter and terminator as effector constructs served as controls in this assay. The transfected protoplasts were treated without (white columns) or with 3 μM ABA (gray columns). The expression of the LUC reporter was normalized to that of the GUS reporter and expressed as fold induction compared to EV in the absence of ABA (1.1 × 10^3^ relative light units per relative fluorescence). Three independent transfections plus three technical replicates per data point, mean ± SD. ***, *P* < 0.001 (one‐way ANOVA); white bars compared to EV without ABA treatment, and black bars compared to EV with ABA treatment. (d–g) Leaf growth and transpiration analyses at 150 μmol m^−2^ s^−1^ photosynthetic active radiation (PAR) and under well‐watered conditions. (d) Rosette pictures (left panel) of 45‐d‐old and representative thermal images (right panel) of 35‐d‐old wt and pGC1:R6 lines. The leaf temperature is shown as false colors indicated on the right panel. (e) Projected leaf area and (f) dry biomass of plants shown on the left panel of (d). (g) Leaf temperature values of plants shown on the right panel of (d). The scale bars in (d) indicate 2 cm. (e, f) *n* = 4 and (g) *n* = 5 biological replicates, mean ± SE. *, *P* < 0.05; **, *P* < 0.01; ***, *P* < 0.001 (one‐way ANOVA) compared to the wt.

We decided to use the reported *pGC1* promoter to drive the expression of ABA receptors in guard cells due to its advantageous tissue specificity and relatively high expression levels over several other guard‐cell promoters (Yang *et al*., 2008). We generated pGC1:GFP‐RCAR6 lines, hereafter simplified as pGC1:RCAR6, as prototypes of guard‐cell ABA receptor overexpressors. The aminoterminally tagged GFP allows a visual inspection of whether the reported *pGC1* promoter restricts RCAR6 expression in guard cells. Two representative pGC1:RCAR6 lines were selected, and homozygous plantlets or seedlings were analyzed for guard cell–specific expression. The GFP signal was exclusively found in guard cells (Fig. 1a), but not in mesophyll cells or root tissues (Fig. S1a,b). Gene expression analysis of isolated guard cells from both pGC1:RCAR6 lines revealed up to 100‐fold elevated RCAR6 transcripts compared to that in the wt plants (Fig. 1b). We next examined whether this expression is able to simulate ABA receptor function of RCAR6 and is sufficient to affect leaf growth and transpiration. Our results indicated that the fusion protein (GFP‐RCAR6) kept the RCAR6 function in inducing ABA‐responsive LUC expression in a transient protoplast assay (Fig. 1c) as reported for RCAR1 (Ma *et al*., 2009). The growth of both pGC1:RCAR6 lines was indistinguishable by visual observation and quantification of leaf area and above‐ground dry biomass from the wt and the p35S:RCAR6 control lines cultivated at a PAR of 150 μmol m^−2^ s^−1^ (Fig. 1d–f). Thermal imaging of leaf rosettes as a proxy for the cooling effect of transpiration revealed up to 0.7°C higher leaf temperature in pGC1:RCAR6 lines than the wt, approaching the temperature of the p35S:RCAR6 line (Fig. 1d,g). These analyses indicated that the *pGC1* promoter is suitable for guard cell–specific expression of an ABA receptor and that the expression level is sufficient to affect transpiration.

The unaltered leaf growth in pGC1:RCAR6 lines is likely because CO_2_ is not a growth‐limiting factor under 150 μmol m^−2^ s^−1^ PAR. To test this possibility, we analyzed the leaf growth and temperature of the better‐performing line, pGC1:RCAR6‐1, under 400 μmol m^−2^ s^−1^ PAR (light saturating point; Yang *et al*., 2016). Again, we could show that, similar to the p35S:RCAR6 line, the pGC1:RCAR6‐1 line displayed > 1°C higher leaf temperature than the wt control (Fig. S2a,b) without growth penalty in rosette size and biomass accumulation (Fig. S2c,d) under higher light conditions.

### Guard‐cell‐specific expression of abscisic acid receptors affects transpiration and growth

**Fig. 2:**
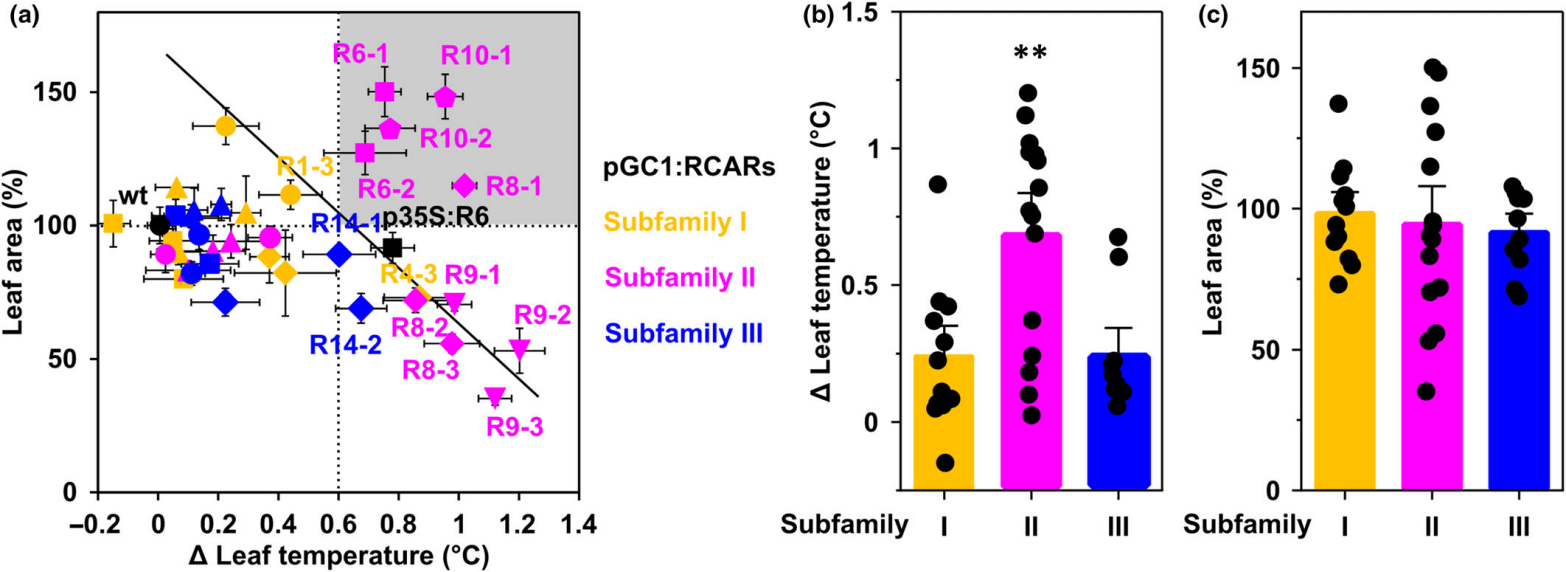
Variation in rosette leaf area and leaf surface temperatures in different Arabidopsis lines with guard cell‐specific expression of green fluorescent protein (GFP)‐tagged abscisic acid (ABA) receptors. (a) Forty‐day‐old wt plants (filled black circles), p35S:R6 line (filled black squares), and lines expressing single subfamily I receptors (RCAR1‐RCAR4, yellow), subfamily II receptors (RCAR5‐RCAR10, red), or subfamily III (RCAR11‐RCAR14, blue) were analyzed with 2 or 3 independent lines per receptor. The leaf area is expressed as a percentage of projected rosette size relative to the wt (37.9 ± 2.6 cm^2^ set to 100%). The leaf temperature is the °C difference compared to the wt (21.3 ± 0.04°C). The trend line for the correlation of leaf growth and temperature increase is depicted using data points shown as labeled symbols with leaf temperature > 0.6°C than the wt (*P* < 0.001, one‐way ANOVA, correlation coefficient *R* = 0.4, *P* = 0.1, linear regression analysis). The dotted lines split the figure into four parts, and the part with gray color highlights guard‐cell receptor overexpressors that combine increased leaf temperature with maintained leaf growth. *n* = 4 biological replicates per data point, mean ± SE. (b) The average difference in leaf temperature (∆*T* in °C, mean ± SE) and (c) leaf area (%) of receptor lines shown in (a) from each subfamily with 12, 15, and 10 independent lines for subfamily I, II, and III receptor lines, respectively, relative to the wt. **, *P* < 0.01 (one‐way ANOVA) compared to the wt.

Based on these promising results, the other ABA receptors were expressed under the control of the *pGC1* promoter accordingly to see whether they could generate the same traits as RCAR6. Analysis of GFP‐tagged representative homozygous lines showed guard cell–specific GFP expression for receptors at subgroup I (RCAR1‐RCAR4), subgroup II (RCAR5‐RCAR7 and RCAR10), and subgroup III (RCAR11‐RCAR14) (Fig. S3). It is noteworthy that pGC1:RCAR8, pGC1:RCAR9, and pGC1:RCAR12 lines displayed GFP signals in pavement cells in addition to guard cells. All receptor overexpressors, including the two pGC1:RCAR6 lines, were then examined for cooling by transpiration and rosette size at 150 μmol m^−2^ s^−1^ PAR. Expression of these receptors in guard cells led to considerable variation in leaf temperature and growth (Fig. 2a). ABA receptors belonging to subfamily II provided on average across the 15 lines analyzed a temperature increase of 0.7 ± 0.1°C (Fig. 2b). By contrast, subfamily I and III lines displayed on average a slight temperature increase of *c.* 0.2°C (Fig. 2b), although expression of RCAR4 belonging to subfamily I and RCAR14 belonging to subfamily III resulted in a leaf temperature increase of above 0.6°C (Fig. 2a). On the other hand, the averaged leaf growth of each of the three subfamily receptors did not differ from the wt (Fig. 2c). In terms of the trait of high WUE without growth penalty, the combination of sustained leaf growth and reduced transpiration is interesting. Hence, the focus was on a leaf temperature increase above 0.6°C and a comparable or larger rosette size than the wt. Only two different receptors provided this trait, receptor RCAR6 and RCAR10 of subfamily II (shaded area in Fig. 2a). Guard‐cell RCAR8 expression in three independent lines elevated the leaf temperature by > 0.8°C, but two lines showed growth reduction (Fig. 2a). A further increase in leaf temperature tended to link to restriction in leaf growth, as illustrated by three pGC1:RCAR9 lines (Fig. 2a).

### Enhanced transient and integrated water use efficiency of pGC1:RCAR6 lines at the cost of reduced carbon assimilation

**Fig. 3:**
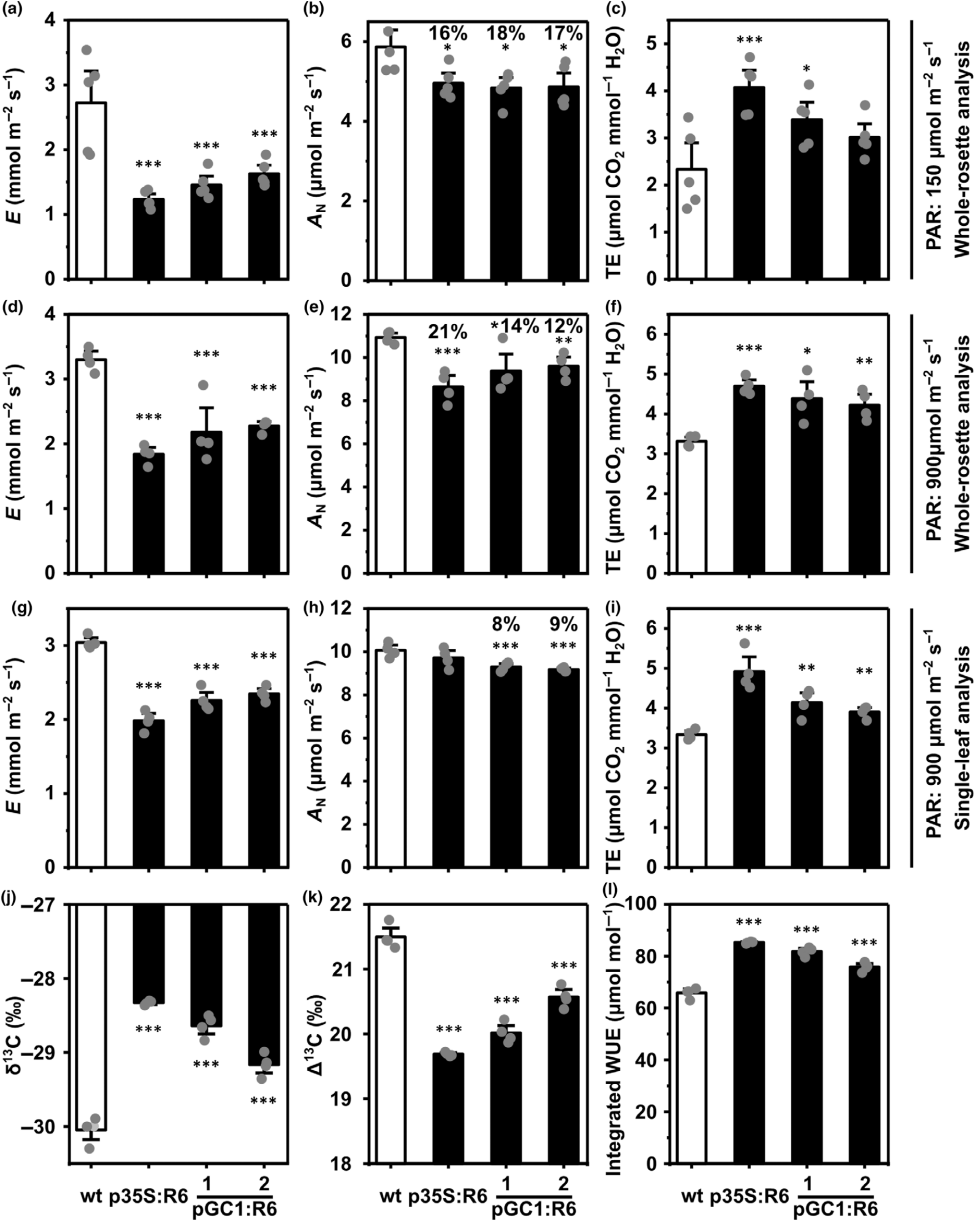
Enhanced leaf‐level transpiration efficiency with reduced net carbon assimilation rate in pGC1:R6 lines. (a–c) Gas exchange analysis was conducted at 150 μmol m^−2^ s^−1^ photosynthetic active radiation (PAR) with whole‐rosettes of Arabidopsis plants. (a) Transpiration rate (*E*), (b) net carbon assimilation rate (*A*_N_), and (c) the derived transpiration efficiency (TE; *A*_N_/*E*) of 31‐d‐old wt (white column), p35S:R6, and pGC1:R6 lines (black columns). (d–f) Gas exchange analysis was conducted at 900 μmol m^−2^ s^−1^ PAR with a whole‐rosette configuration. (c) *E*, (d) *A*_N_, and (e) TE of 40‐d‐old wt, p35S:R6, and pGC1:R6 lines. (g–i) Gas exchange analysis was conducted at 900 μmol m^−2^ s^−1^ PAR with a single leaf (leaf number 16 ± 3, 2 wk after leaf emergence) clamped in a 2 cm^2^ cuvette. (g) *E*, (h) *A*_N_, and (i) TE of the wt, p35S:R6, and pGC1:R6 lines. (j) ^13^C composition (δ^13^C), (k) ^13^C discrimination (Δ^13^C), and (l) estimated integrated water use efficiency (WUE) of leaf dry materials of the wt (white column), p35S:R6, and pGC1:R6 lines (black columns) shown in Fig. 1(f). (b, e, h) The numbers above the columns indicate reduction in *A*_N_ compared to the wt. (a–c) *n* = 5 and (d–l) *n* = 4 biological replicates, mean ± SE; *, *P* < 0.05; **, *P* < 0.01; ***, *P* < 0.001 (one‐way ANOVA) compared to the wt.

Both ABA receptors (RCAR6 and RCAR10) that provided a consistent trait of high WUE without trade‐offs in leaf growth by guard cell–specific expression (Fig. 2a) were previously identified for this trait under a cell‐nonselective promoter (Yang *et al*., 2016). These lines now allow a physiological analysis to separate enhanced ABA signaling in stomata from other cells and plant tissues. We chose the pGC1:RCAR6 lines for closer characterization because most physiological analyses have been carried out previously with the p35S:RCAR6 line (Yang *et al*., 2016, 2019). By analyzing the gas exchange using a whole‐rosette configuration with 150 μmol m^−2^ s^−1^ PAR, we could show that pGC1:RCAR6 lines reduced E by > 40% compared to the wt, somewhat lower than the reduction in the p35S:RCAR6 line (54%; Fig. 3a). Reduced leaf transpiration points to a stomatal limitation for water. As expected, similar to the p35S:RCAR6 line, both pGC1:RCAR6 lines showed up to 42% lower *g*_s_ compared to the wt (Fig. S4a).

A stomatal limitation also restricts CO_2_ entry for photosynthesis, and our result indicated a *c*. 17% reduction in *A*_N_ for the p35S:RCAR6 and pGC1:RCAR6 lines compared to the wt (Fig. 3b). Consequently, the disproportional reduction in *A*_N_, *E*, and *g*_s_ led to a respective > 30 and 28% increase in transpiration efficiency (TE, *A*_N_/*E*) and iWUE for pGC1:RCAR6 lines than that in the wt, somewhat underperforming relative to the p35S:RCAR6 line (Figs 3c, S4b). Comparable results were observed at saturating light conditions of 900 μmol m^−2^ s^−1^ PAR under which CO_2_ is a limiting factor for whole Arabidopsis rosettes (Figs 3d–f, S4c,d) and single leaves (Figs 3g–i, S4e,f). While the difference in *A*_N_ of the p35S:RCAR6 line lay between significant and non‐significant levels, depending on the experimental setup, *A*_N_ of the pGC1:RCAR6 lines and *E* and leaf‐level WUE of RCAR6 transgenic lines consistently showed significant differences, with the pGC1:RCAR6 lines again slightly underperforming compared to the p35S:RCAR6 line.

The observed stomatal limitations in the overexpressors can be attributed to changes in stomatal development and regulation of aperture. Compared to the wt, stomatal density was reduced by up to 23% in guard‐cell overexpressors, and by 27% in the p35S:RCAR6 line (Fig. S5a). Stomatal size slightly increased by *c*. 5% in p35S:RCAR6 and pGC1:RCAR6‐1, and by 11% in pGC1:RCAR6‐2 (Fig. S5b). Analysis of stomatal aperture revealed a consistent reduction of *c*. 25% across both p35S:RCAR6 and pGC1:RCAR6 lines (Fig. S5c).

Besides a stomatal limitation to CO_2_ conductance, *A*_N_ can be affected by biochemical constraints of the carboxylation rate of ribulose‐1,5‐bisphosphate carboxylase/oxygenase (Rubisco) and the rate of RuBP regeneration (Farquhar *et al*., 1980). By comparing *A*_N_ at varying intercellular CO_2_ (C_i_) levels (*A*_N_–C_i_ curves), we could rule out this possibility because no significant difference in *A*_N_ between the p35S:RCAR6 line or pGC1:RCAR6 lines and the wt was observed at variable C_i_ (Fig. S6; Table S3). Consistently, the maximum PSII efficiency under dark and PSII operating efficiency determined under illumination were comparable among different genotypes (Fig. S7a–d).

While TE and iWUE provide short‐term readouts for carbon‐to‐water efficiency, carbon isotope discrimination (Δ^13^C) in plant dry biomass offers an integrated measure of WUE over time (Farquhar *et al*., 1989). Analysis of ^13^C composition (δ^13^C) in aboveground dry biomass of plants grown at 150 and 400 μmol m^−2^ s^−1^ PAR showed up to 1.4 and 2.2‰ higher δ^13^C in pGC1:RCAR6 lines compared to the wt, respectively, approaching values of corresponding p35S:RCAR6 controls (Figs 3j, S8a). The δ^13^C values allow for deduction of Δ^13^C and thereafter an integrated WUE, which indicated lower ^13^C discrimination (Figs 3k, S8b) and up to 24 and 45% higher integrated WUE for the pGC1:RCAR6 lines than the wt at both light intensities, respectively, slightly lower than the p35S:RCAR6 line (Figs 3l, S8c). Taken together, activation of ABA signaling in guard cells by RCAR6 enhances transient and long‐term leaf WUE but negatively affects *A*_N_ because of stomatal limitation.

### Increased leaf water potential and turgor in pGC1:RCAR6 lines

**Fig. 4:**
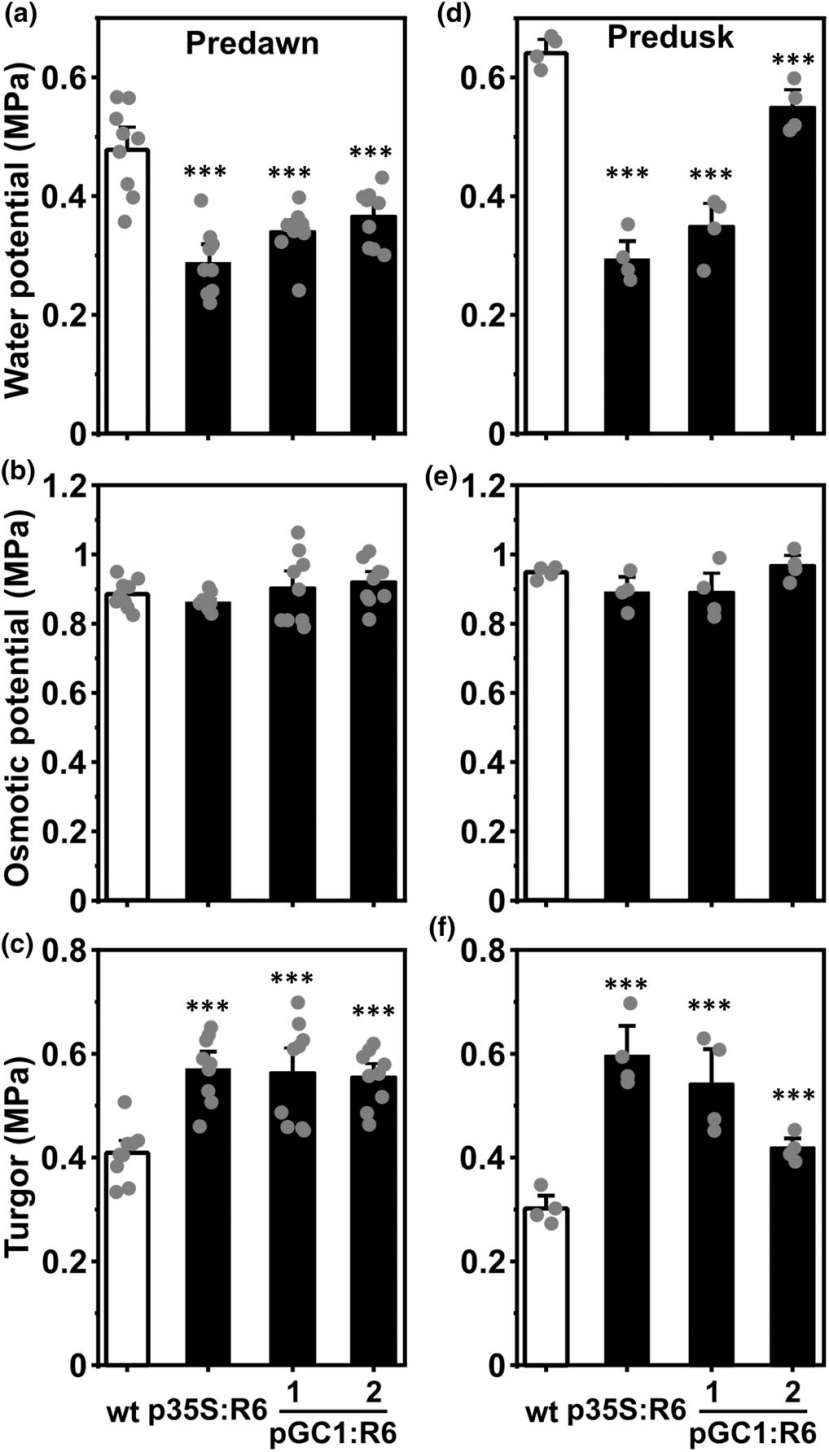
Increased leaf water potential conferred by R6 expression in guard cells. (a) Predawn leaf water potential, (b) osmotic potential, (c) turgor, and (d) predusk water potential, (e) osmotic potential, (f) and turgor of the Arabidopsis wild‐type (wt, white columns), p35S:R6, and pGC1:R6 lines (black columns). The leaf turgor was derived from the difference between leaf water potential and osmotic potential. (a–f) All plants were grown under 8 h : 16 h, day : night photoperiods (day‐night light intensity, temperature, and relative humidity: 150/0 μmol m^−2^ s^−1^, 22/17°C, and 50/60%) and well‐watered conditions for 58–62 d, and the 24^th^–26^th^ leaf of each genotype was analyzed. (a–c) *n* = 9, (d–f) *n* = 4 biological replicates, mean ± SE, ***, *P* < 0.001 (one‐way ANOVA) compared to the wt.

Apparently, *A*_N_ is insufficient to explain the leaf growth of pGC1:RCAR6 lines, suggesting the presence of other mechanisms. One such mechanism may be implicated in an increased turgor and water potential in the leaves. Hence, the leaf water potential and osmotic potential were determined by using the pressure chamber technique and osmometer, respectively. At predawn, we could show that the water potential was less negative in the p35S:RCAR6 line and pGC1:RCAR6 lines, while the osmotic potential was comparable to the wt (Fig. 4a,b). The resulting leaf turgor, as the difference between leaf water potential and osmotic potential, increased from 0.4 MPa in the wt to *c*. 0.55 MPa in the p35S:RCAR6 line and pGC1:RCAR6 lines, respectively (Fig. 4c). At predusk, the water potential decreased in wt and pGC1:RCAR6‐2 compared to that at predawn, while the water potential of the p35S:RCAR6 line and pGC1:RCAR6‐1 was unaltered (Fig. 4d). The osmotic potentials did not differ significantly from predawn values (Fig. 4e). Consequently, the turgor dropped to 0.3 and 0.4 MPa in the wt and pGC1:RCAR6‐2, respectively, but stayed above 0.5 MPa in the p35S:RCAR6 and the pGC1:RCAR6‐1 lines (Fig. 4f). Our results support an increase in leaf water potential of *c*. 0.15–0.2 MPa by ectopic expression of RCAR6 and unchanged osmotic potentials resulting in a higher cell turgor.

### Effect on leaf size by ectopic RCAR6 expression in guard cells

**Fig. 5:**
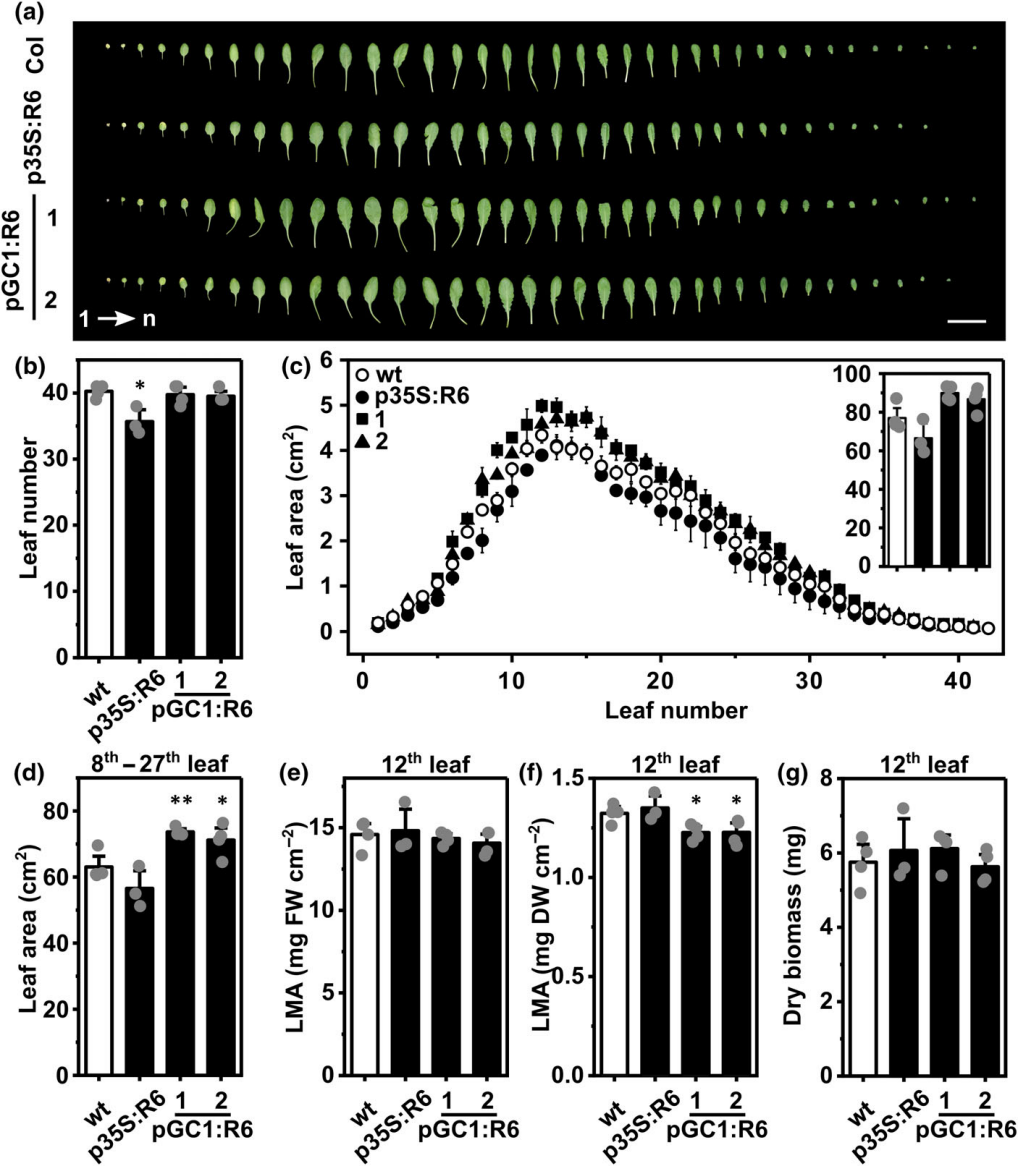
Overexpressing RCAR6 in guard cells enlarges leaf area. (a) Representative pictures of individual leaves of 57‐d‐old Arabidopsis wild‐type (wt), p35S:R6, and pGC1:R6 lines were aligned according to their sequence of emergence (1 to *n* from the left panel to the right as indicated by the arrow). Bar, 2 cm, (b) total leaf number (wt, white column; p35S:R6 and pGC1:R6 lines, black columns), and (c) individual leaf size (wt, open circles; p35S:R6, filled circles; pGC1:R6‐1, filled rectangles; pGC1:R6‐2, filled triangles) in the experiment as shown in (a). The inset in (c) indicates the total leaf area of different genotypes. (d) The sum of the area of the 8^th^ to the 27^th^ leaf is shown in (c). Leaf mass per area (LMA) on (e) the fresh‐weight basis and (f) the dry‐weight basis and (g) the leaf dry biomass of the 12^th^ leaf of the wt (white columns), p35S:R6, and pGC1:R6 lines (black columns). (e–g) The midvein of the 12^th^ leaf was not taken into account for assessing the leaf thickness but was included in determining the whole‐leaf biomass. (a–g) Each leaf was tracked, numbered, excised, and aligned according to its order of emergence. *n* = 4 biological replicates, mean ± SE. *, *P* < 0.05; **, *P* < 0.01 (one‐way ANOVA) compared to the wt.

In the light of improved water status in the p35S:RCAR6 and pGC1:RCAR6 lines, the growth of individual leaves was more closely examined (Fig. 5). Alignment of the leaves from the 57‐d‐old and nonbolting plants grown at 150 μmol m^−2^ s^−1^ PAR revealed fewer leaves in the p35S:RCAR6 line compared to the wt, while no difference in leaf numbers was observed in the pGC1:RCAR6 lines (Fig. 5a,b). However, the trend of a larger intermediate‐aged leaf area in the pGC1:RCAR6 lines was visible (Fig. 5c) compared to the wt but not for the p35S:RCAR6 line, which was significant among the larger leaves from position 8 to 27 (Fig. 5d). The leaf thickness as exemplified for the 12^th^ leaf and expressed as LMA was similar among different genotypes on a fresh weight basis (Fig. 5e) but was by *c*. 7% lower on a dry weight basis for the pGC1:RCAR6 lines (Fig. 5f). The lamina of the 12^th^ leaf in the pGC1‐RCAR6 lines was on average (*n* = 8) increased by 10% compared to the wt (Fig. 5c) while the leaf biomass was similar (Fig. 5g).

### Enhanced drought resistance by ectopic RCAR6 expression in guard cells

**Fig. 6:**
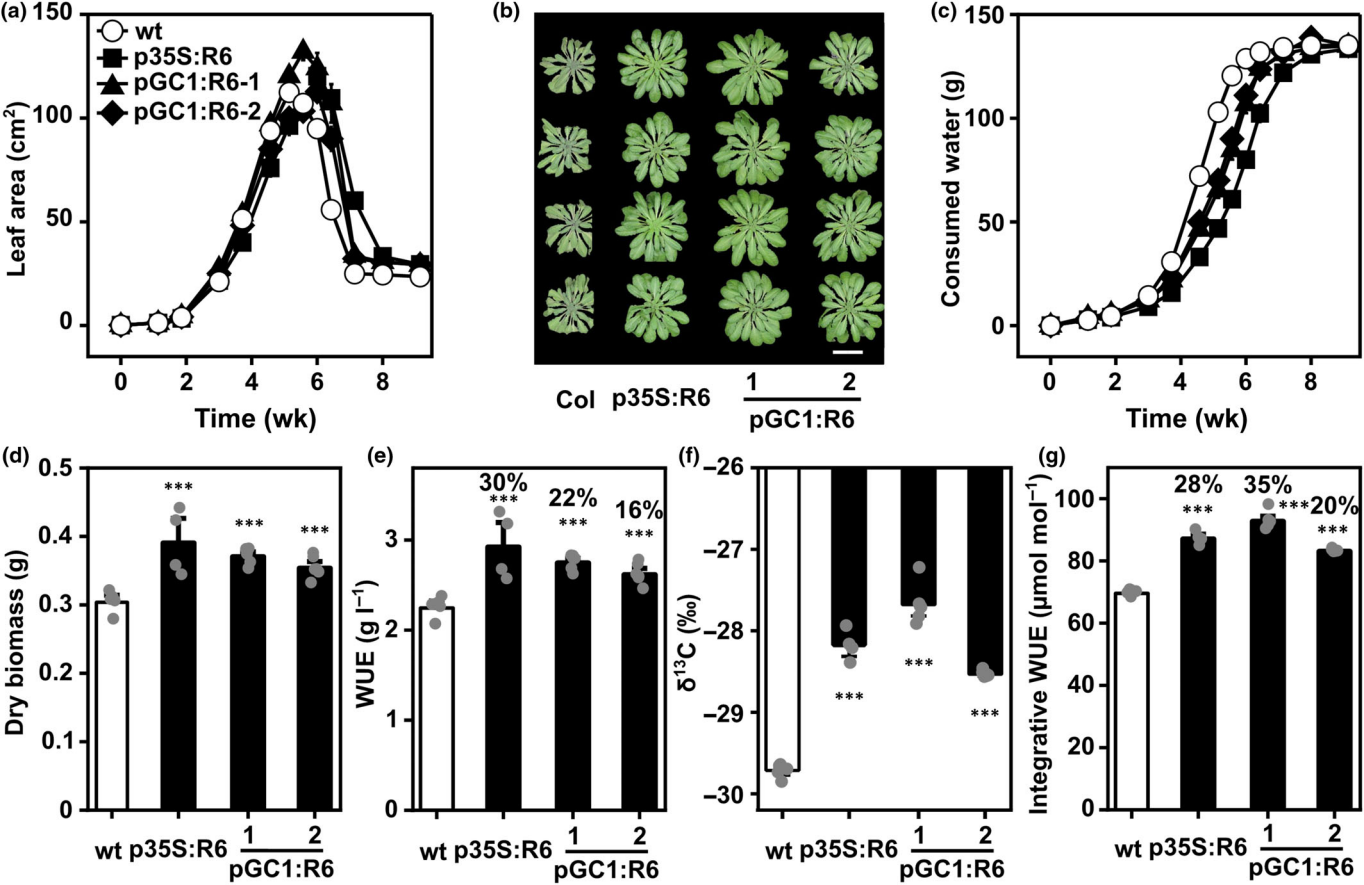
Enhanced drought resistance and water use efficiency (WUE) conferred by guard‐cell expression of RCAR6 under progressive drought. (a–g) Single Arabidopsis plantlets were allowed to grow for 18 d under well‐watered conditions before discontinuation of watering. The soil surface was covered to prevent evaporation. All water in the soil was consumed via leaf transpiration, therefore exerting a developed water deficit. (a) Kinetics of leaf growth of the wild‐type (wt, open circles), p35S:R6 (filled squares), and pGC1:R6‐1 (filled triangles) and pGC1:R6‐2 (filled diamonds) over the time of progressive drought. The projected leaf area was used as an indicator of leaf growth. The reduced leaf area was due to leaf wilting. (b) Wilted wt plants and turgescent leaf rosettes of p35S:R6 and pGC1:R6 lines at day 39. All plants were randomized during the experiment. Pictures of rosettes were aligned according to their genotype after imaging. Bar, 2 cm. (c) Kinetics of consumed water of the wt (open circles), p35S:R6 (filled squares), and pGC1:R6‐1 (filled triangles) and pGC1:R6‐2 (filled diamonds) in the progressive drought. At day 75, the rosettes were harvested. (d) The aboveground dry biomass, (e) biomass‐derived WUE (ratio of biomass to consumed water), (f) δ^13^C of leaf dry materials in (d, g) ^13^C‐derived integrated WUE of the wt (white columns), p35S:R6, and pGC1:R6 (black columns) were determined. (a–g) *n* = 4 biological replicates, mean ± SE, ***, *P* < 0.001 (one‐way ANOVA) compared to the wt.

The pGC1:RCAR6 lines were subjected to progressive drought to investigate their resilience under water limitation at 150 μmol m^−2^ s^−1^ PAR. The plants were grown without further watering in pots containing water‐saturated soil that were covered to prevent evaporation. The initial SWC was identical in all samples. Plant growth was expressed as an increase in the projected leaf area of the rosettes, which reached a maximum after *c*. 5 wk (Fig. 6a). Maximum rosette size was 112 ± 3 cm^2^ for the wt at day 36, whereas the other lines grew further and reached the maximum at day 42, 39, and 42 with a size of 120 ± 4, 132 ± 4, and 113 ± 3 cm^2^ for the p35S:RCAR6 line and two pGC1:RCAR6 lines, respectively. At day 42, the leaves of the p35S:RCAR6 line and pGC1:RCAR6 lines were still turgid but wilted in wt plants, and their rosettes were shrunk (Fig. 6b). The postponement of wilting reflected the slower rate of water consumption by RCAR6 expression (Fig. 6c). After the consumption of all plant‐available water at day 64, the wt yielded 0.3 ± 0.01 g aboveground dry biomass, whereas the p35S:RCAR6 line produced on average 30% more aboveground dry biomass, and pGC1:RCAR6 lines showed up to a 22% increase compared to the wt (Fig. 6d). Since all lines consumed the same amount of water, the higher biomass accumulation in the pGC1:RCAR6 lines showed up to a 22% higher aboveground biomass‐based WUE, similar to that of the p35S:RCAR6 line (Fig. 6e). Further assessment revealed a higher ^13^C composition and less ^13^C discrimination of the p35S:RCAR6 line than the wt and a 28% increased integrated WUE, and such performance in pGC1:RCAR6 lines was comparable to or even better than the RCAR6 line (Fig. 6f,g).

We next investigated the trait of high WUE without trade‐offs in leaf growth under controlled water deficit. We grew plants under controlled SWC conditions at *c*. 60% (85% field capacity; well watered) and 20% (moderately severe drought). After 5 wk treatment, water consumption by the p35S:RCAR6 and pGC1:RCAR6 lines was reduced by 30, 15, and 20%, respectively, compared to the wt (152.4 ± 4.5 g) at 60% SWC. Consumed water under water deficit was reduced by 26, 16, and 11% lower than that of the wt (94.5 ± 0.5 g) at 20% SWC (Fig. S9a). The biomass was reduced in all genotypes under drought compared to that under well‐watered conditions. Both the p35S:RCAR6 and guard cell‐overexpressors maintained biomass levels comparable to the wt under well‐watered and water‐deficit conditions, indicating no apparent growth trade‐offs (Fig. S9b). WUE was significantly enhanced in the pGC1:RCAR6 lines, with up to 40 and 20% under well‐watered and water‐deficit conditions, respectively, compared to the wt (Fig. S9c). These results indicated that pGC1:RCAR6 lines mimicked the p35S:RCAR6 line, being more water‐use efficient without trade‐offs in growth under drought.

## Discussion

ABA is widely regarded as a stress phytohormone that prepares plants for harsh environmental conditions. An apparent role of ABA is reflected in the induction of stomatal closure and the inhibition of growth. In this study, we provide new insights into the interlaced roles of guard‐cell ABA signaling, leaf transpiration, leaf water status, and growth, which contribute to high plant WUE without trade‐offs in growth.

### Activation of specific abscisic acid signaling in guard cells does not necessarily result in a leaf growth penalty

Our results demonstrated that guard cell‐specific expression of subfamily II ABA receptors, particularly RCAR6, RCAR8, RCAR9, and RCAR10, had a greater effect on reducing leaf transpiration than expression of receptors from subfamilies I and III (Fig. 2a,b). Notably, RCAR14, a subfamily III member, also strongly reduced transpiration (Fig. 2a). Previous high‐order mutant analyses identified RCAR3, RCAR8, RCAR10, RCAR11, RCAR12, and RCAR14 as functionally involved in stomatal regulation under various environmental conditions (Gonzalez‐Guzman *et al*., 2012; Dittrich *et al*., 2019). Interestingly, RCAR8 and RCAR10 complemented defects in CO_2_‐induced stomatal closure in the *rcar11;rcar14;rcar10;rcar8;rcar3* quintuple mutant, while RCAR14 restored impaired ABA‐induced stomatal closure (Dittrich *et al*., 2019), suggesting that endogenous expression of these three receptors is essential for proper stomatal regulation under elevated CO_2_ and drought conditions. Among these three receptors, RCAR10 exhibited the highest expression in guard cells, whereas RCAR8 showed much lower expression, *c*. 1/25 of RCAR10 (Fig. S10). The relatively low abundance of RCAR8 may be compensated by its high ABA‐binding affinity (Okamoto *et al*., 2013).

By contrast, RCAR6 and RCAR9 function was revealed only through overexpression studies, suggesting that transgenic expression may override native tissue‐specific or quantitative constraints. Indeed, both receptors are only weakly expressed in guard cells (Fig. S10; Yang *et al*., 2008). Although RCAR6 and RCAR9 differ substantially in ABA‐binding affinity (Okamoto *et al*., 2013), both can induce ABA‐responsive gene expression in mesophyll protoplasts (Fig. 1c; Tischer *et al*., 2017). Given that core ABA signaling components are largely conserved across cell types, it is plausible that strong overexpression of both receptors enhances basal ABA sensitivity in guard cells by promoting the inhibition of PP2Cs, especially ABI1 and ABI2 (Dittrich *et al*., 2019; Yang *et al*., 2019), thereby sensitizing the stomatal response to ABA. These results illustrate how structurally related ABA receptors can regulate stomatal function through different combinations of ABA affinity, expression level, and tissue specificity. Although RCAR6 and RCAR9 have not been explicitly linked to CO_2_ or other environmental cues, their capacity to enhance ABA sensitivity suggests broader regulatory potential. Further studies are needed to clarify their roles beyond canonical ABA signaling.

We further showed that the reduced transpiration observed in pGC1:RCAR6 lines was attributed to reduced stomatal density and aperture (Fig. S5a,c). While ABA is known to target SnRK2.2, 2.3, and 2.6 to suppress SPEECHLESS and MUTE for delaying stomatal development (Tanaka *et al*., 2013; Mohamed *et al*., 2023), our results suggest that this regulation can occur in a guard‐cell‐autonomous manner and not only controls aperture but also influences stomatal patterning.

Although overexpressors of RCAR6, RCAR8, RCAR9, and RCAR10 all showed dramatically increased leaf temperature, they displayed considerable variation in leaf growth depending on receptors (Yang *et al*., 2019). Interestingly, the phenotypes observed in non‐tissue‐specific receptor overexpressors were recapitulated in guard cell‐specific overexpression lines (Fig. 2a). Independent guard‐cell RCAR6 and RCAR10 overexpressors behaved consistently in reducing leaf transpiration while maintaining leaf growth (Figs 1d–f, 2a), suggesting that the high WUE without trade‐offs in leaf growth is from their expression in guard cells. By contrast, pGC1:RCAR8 and pGC1:RCAR9 lines strongly reduced leaf growth (up to 75%) while keeping low transpiration compared to the wt (Fig. 2a). One exception was observed among pGC1:RCAR8 lines, showing leaf temperature slightly higher than the other two independent pGC1:RCAR8 lines but with uncompromised leaf growth (Fig. 2a), which may result from disturbance of the endogenous gene function at the insertion site of the transgene. The growth penalty observed in pGC1:RCAR8 and pGC1:RCAR9 lines is unlikely to stem from receptor activity alone (Tischer *et al*., 2017) and may also be attributed to their expression levels in guard cells and expression leakage into pavement cells (Fig. S3). The mechanisms underlying this leakage remain unclear, but the overproduction of RCAR8 and RCAR9 may exceed the cellular degradation capacity (Fernandez *et al*., 2020; Vieira *et al*., 2025). ABA‐inhibited pavement cell expansion (den Os *et al*., 2007; Savaldi‐Goldstein *et al*., 2007) may explain the associated growth arrest.

pGC1:RCAR6 enhanced WUE without growth penalty also under two different water‐deficit scenarios (Figs 6c–e, S9). There was an advantage of biomass accumulation under progressive drought, which is due to their higher intrinsic WUE, particularly during the well‐watered phase, as demonstrated in our previous study using the p35S:RCAR6 line (Yang *et al*., 2016). These findings highlight the pivotal role of specific ABA signaling pathways and guard‐cell expression in achieving high WUE without compromising leaf growth and providing new revenue for future biochemical, biotechnological, and breeding efforts to develop water‐saving and high‐WUE crops without trade‐offs in growth and productivity.

### Abscisic acid‐induced stomatal closure targets leaf water to maintain leaf biomass accumulation

Previous work using the p35S:RCAR6 overexpression line showed that the reduction in *g*_s_ and the resulting enhancement in WUE were most prominent under well‐watered conditions, with the differences diminishing during progressive drought stress (Yang *et al*., 2019). These findings suggest that guard‐cell ABA signaling primarily tunes water‐use traits when water is not limited, and that its impact is less pronounced as soil moisture declines. Based on this, our current study focuses on well‐watered conditions to uncover the physiological mechanisms by which reduced *g*_s_ and maintained growth contribute to enhanced WUE in pGC1:RCAR6 lines.

It is well‐established that leaf growth positively correlates with *A*_N_, and the latter was positively associated with *g*_s_, given that RuBP regeneration is not limited (Wong *et al*., 1979; Shipley & Vu, 2002). Accordingly, transgenic plants with an increased *g*_s_ resulting from guard‐cell expression of a proton ATPase (AHA2) displayed a concurrent increase in *g*_s_, *A*_N_, and growth compared to the wt control (Wang *et al*., 2014). In pGC1:RCAR6 lines with reduced *g*_s_, *A*_N_ had declined compared to the wt (Figs 3b,e,h, S4a,c,e), and according to our data, the reduction of *A*_N_ is due to the stomatal limitation rather than any biochemical constraints (Figs S6, S7). *A*_N_ could, however, also be constrained by nonoptimal mesophyll CO_2_ diffusion. In our study, a reduced leaf thickness in pGC1:RCAR6 lines comes with a reduced leaf dry biomass per area, indicative of thinner cell walls or/and less densely packed mesophyll cells which all may shorten the CO_2_ diffusion path to carboxylation sites. However, as seen with an apparently reduced *A*_N_, this potentially more efficient mesophyll CO_2_ diffusion in pGC1:RCAR6 lines does not entirely compensate for the observed significantly reduced stomatal CO_2_ influx (Fig. 3b,e,h). Provided assimilated CO_2_ is the only source for leaf growth, a 17% reduction in *A*_N_ of pGC1:RCAR6 lines would undoubtedly result in a reduction in the accumulation of leaf dry biomass after 45‐d growth compared to the wt. This inconsistency in simulated and actual leaf growth clearly reflects a disassociation of *A*_N_ with leaf growth (Figs 1e,f, 3b,e,h, S2e). Such a decoupling of *A*_N_ with leaf growth was also reported for germplasms with altered ABA sensitivity (Fujii *et al*., 2009; Zheng *et al*., 2010; Pizzio *et al*., 2024) and stomatal development (Masle *et al*., 2005; Hughes *et al*., 2017). To reconcile the observation of reduced *g*_s_ and *A*_N_ with uncompromised leaf growth, there must be other growth‐promoting mechanisms compensating for growth inhibition caused by reduced *A*_N_.

One of the mechanisms is implicated in leaf water status. Three observations suggested an improved leaf water status in pGC1:RCAR6 lines compared to the wt. First, leaf transpiration of pGC1:RCAR6 lines was reduced compared to the wt (Figs 1d,g, 3a,d,g, S2a,b). Second, the leaf water content (deduced from the difference between fresh‐biomass and dry‐biomass derived LMA) of the 12^th^ leaf of pGC1:RCAR6 lines and the water potential of the 24^th^ to 26^th^ leaves in pGC1:RCAR6 lines were higher than in the wt (Figs 4a,d, 5e,f). These observations align with previous findings (Ache *et al*., 2010) and support a link between reduced transpiration and elevated leaf water potential mediated by guard cell–specific ABA signaling. The third observation is the slightly thinner leaf (dry biomass‐derived LMA) of the 12^th^ leaf in pGC1:RCAR6 lines than that of the wt (Fig. 5f). Leaf thickness has been suggested to be associated with leaf water status, with thinner leaves typically observed in non‐water‐deficit habitats and thicker leaves in water‐scarce environments (Poorter *et al*., 2009).

An improved leaf water status in pGC1:RCAR6 plants was associated with enhanced expansive growth of intermediate‐aged leaves, exemplified by the larger 8^th^ to 27^th^ leaf (Fig. 5d). These leaves represent over 50% of the total leaf area (Fig. 6c,d) and are likely the most photosynthetically active portion of the rosette. Given their dominance in total leaf area and photosynthetic contribution, these intermediate‐aged leaves can be considered representative of the average photosynthetic performance of the whole rosette. Notably, while the *A*_N_ (leaf‐area‐normalized) was reduced by *c*. 17% in the pGC1:RCAR6 lines (Fig. 3b), the intermediate‐aged leaf area increased by *c*. 10% (Fig. 5d), effectively mitigating the decline in *A*_N_. This compensatory increase in photosynthetically active surface area likely sustains whole‐leaf carbon assimilation, ultimately maintaining overall leaf biomass accumulation.

Consistently, Salah & Tardieu (1997) showed a concurrent diurnal variation of leaf expansion rate and water potential, and found that reduced leaf growth correlates with lowered leaf water potential throughout the progressive drought. In line with our observation, excessive transpirational water loss in transgenic lines in which ABA signaling was specifically repressed in guard cells resulted in arrested leaf growth (Yaaran *et al*., 2019). Notably, p35S:RCAR6 exhibited a leaf water potential comparable to pGC1:RCAR6‐1, but leaf growth of the p35S:RCAR6 line underperformed that of the pGC1:RCAR6 line (Figs 1d–f, 2a and 5c), indicating that RCAR6 expression in non‐guard‐cell tissues negatively impacts leaf growth. Further studies are required to elucidate the underlying mechanisms.

The increased leaf water content and potential in pGC1:RCAR6 lines likely result from an overall effect of reduced leaf transpiration and unimpaired plant hydraulic conductivity. Based on Fick's diffusion law, plant hydraulic conductivity can be estimated as the ratio of E to the water potential difference between the soil and leaf tissue. Under well‐watered conditions (soil water potential *c*. −0.08 MPa; Yang *et al*., 2019), a 47 and 40% reduced E (Fig. 3a) and a 50 and 18% reduced soil‐to‐leaf water potential gradient (calculated as the difference between soil water potential and predusk leaf water potential shown in Fig. 4d) suggest an unaltered or increased plant hydraulic conductivity for pGC1:RCAR6‐1 and pGC1:RCAR6‐2, respectively. The leaf hydraulic conductivity may have a similar tendency to the whole‐plant hydraulic conductivity, assuming a comparable xylem water potential in guard‐cell RCAR6 overexpressors and the wt. While ABA is known to reduce leaf water potential and hydraulic conductivity (Pantin *et al*., 2013; Yaaran *et al*., 2023), these effects can only be primarily attributed to ABA action in tissues other than guard cells, such as the mesophyll and bundle sheath cells (Negin *et al*., 2019; Yaaran *et al*., 2023). These findings support a tissue‐specific role for ABA in regulating leaf hydraulics and water status.

### Improving leaf water status facilitates generating crops combining high water use efficiency without trade‐offs in productivity

Our findings tentatively explain the elusive relationship between WUE and plant growth. TE and iWUE derived from gas exchange parameters and integrated WUE derived from ^13^C techniques have been widely used as probes to breed high‐WUE crops. Both techniques basically detect the exchange efficiency of CO_2_ to water vapor via stomatal pores. However, in practice, WUE refers to the ratio of biomass to consumed water (Blum, 2009). The contribution of leaf water status to leaf growth is taken into the definition of biomass‐based WUE but not considered in the ^13^C technique and the gas exchange approach, and thereby, it is not surprising that high‐WUE crops screened by the advanced approaches have elusive consequences in growth and yield (Blum, 2009; Bhaskara *et al*., 2022). Hence, we propose that the ^13^C technique and gas exchange analysis combined with growth evaluation probably help generate high‐WUE crops without trade‐offs in productivity that are more compatible with the interest of agriculture. The genetic components responsible for the trait of high‐WUE without trade‐offs in productivity involve genes specifically regulating stomatal closure (Figs 1, S2), stomatal development (Dunn *et al*., 2019), and potential genes controlling plant hydraulics.

## Competing interests

None declared.

## Author contributions

ZY conceived and designed different parts of the research. RS, AC and MA supervised different parts of the experiments. JL generated the guard‐cell receptor overexpressors and conducted gas exchange measurements, thermal imaging, PAM imaging, and growth analysis together with ZY. JL carried out leaf water potential, osmotic potential measurements, and confocal microscopy with contributions from ZY, carbon isotope analysis with contributions from RS, and deficit irrigation with contributions from MA. Experiments related to stomatal development and regulation of aperture were carried out by JL. JL and ZY analyzed the data and co‐authored the manuscript with input from all the authors. All authors reviewed and approved the manuscript.

## Disclaimer

The New Phytologist Foundation remains neutral with regard to jurisdictional claims in maps and in any institutional affiliations.

## Supporting information


**Fig. S1** The absence of GFP‐R6 fusion protein in mesophyll and root cells.
**Fig. S2** Reduced leaf transpiration and sustained leaf growth in the pGC1:R6 line at 400 μmol m^−2^ s^−1^ PAR.
**Fig. S3** Guard cell expression of ABA receptors driven by *pGC1* promoter.
**Fig. S4** Reduced stomatal conductance and enhanced intrinsic WUE in pGC1:R6 lines.
**Fig. S5** Altered stomatal development and aperture in pGC1:R6 lines.
**Fig. S6** The uncompromised *A*_N_ at varying intercellular CO_2_ levels in pGC1:R6.
**Fig. S7** Overexpressing RCAR6 in guard cells does not affect photosynthetic apparatus.
**Fig. S8** Enhanced WUE of the pGC1:R6 line growth at 400 μmol m^−2^ s^−1^ PAR.
**Fig. S9** Maintained biomass, reduced water consumption, and enhanced WUE of pGC1:RCAR6 lines under controlled water deficit conditions.
**Fig. S10** Variation in transcript abundance of RCAR6 (R6), RCAR8 (R8), and RCAR10 (R10) in wild‐type guard cells.

**Table S1** Primes used to amplify the *pGC1* promoter and each of the 14 ABA receptors.
**Table S2** Primes employed for RT‐qPCR analysis of target and reference transcripts.
**Table S3** Summary of statistical significance of net carbon assimilation rate (*A*_N_) at each ambient CO_2_ levels (C_a_).
Please note: Wiley is not responsible for the content or functionality of any Supporting Information supplied by the authors. Any queries (other than missing material) should be directed to the *New Phytologist* Central Office.

## Data Availability

Confocal images are presented in Figs S1 and S3; thermal imaging, leaf growth, and ^13^C stable isotope data under well‐watered and high‐light conditions in Figs S2 and S8. Gas exchange values are available in Figs S4 and S6; stomatal density, size, and aperture data in Fig. S5. PAM imaging and values of quantum yield are shown in Fig. S7. Deficit irrigation and receptor expression data are included in Figs S9 and S10, respectively. Primer sequences used to clone the *pGC1* promoter and all 14 ABA receptors are provided in Table S1, and gene identifiers are listed in Table S1. Primer sequences used to detect transcripts of RCAR6, RCAR8, RCAR10, GC1, and UBQ10 (AT4G05320) are listed in Table S2. Statistical significance outcomes for the gas exchange analysis in Fig. S6 are summarized in Table S3. All other data supporting the findings of this study are included within the paper. Transgenic Arabidopsis lines overexpressing ABA receptors are available from the corresponding author upon reasonable request.
