# Supplementary material for: Guard‐cell expression of abscisic acid receptors for engineering water‐use‐efficient plants without trade‐offs in growth
Source: New Phytol. 2025 Jul 24;248(2):690–705. doi: 10.1111/nph.70404 (PMC12445875; doi:10.1111/nph.70404)
Supplement: Supplementary file 1 — Fig. S1 The absence of GFP‐R6 fusion protein in mesophyll and root cells. Fig. S2 Reduced leaf transpiration and sustained leaf growth in the pGC1:R6 line at 400 μmol m−2 s−1 PAR. Fig. S3 Guard cell expression of ABA receptors driven by pGC1 promoter. Fig. S4 Reduced stomatal conductance and enhanced intrinsic WUE in pGC1:R6 lines. Fig. S5 Altered stomatal development and aperture in pGC1:R6 lines. Fig. S6 The uncompromised A N at varying intercellular CO2 levels in pGC1:R6. Fig. S7 Overexpressing RCAR6 in guard cells does not affect photosynthetic apparatus. Fig. S8 Enhanced WUE of the pGC1:R6 line growth at 400 μmol m−2 s−1 PAR. Fig. S9 Maintained biomass, reduced water consumption, and enhanced WUE of pGC1:RCAR6 lines under controlled water deficit conditions. Fig. S10 Variation in transcript abundance of RCAR6 (R6), RCAR8 (R8), and RCAR10 (R10) in wild‐type guard cells. [file NPH-248-690-s001.pdf]

New Phytologist **Supporting Information**

Article title: Guard-cell expression of abscisic acid receptors for engineering water-use-efficient plants without trade-offs in growth

Authors: Jinghui Liu[1], Rudi Schäufele[2], Alexander Christmann[1,3], Mutez Ahmed[3], Zhenyu Yang[1,3]✉

[1]Chair of Botany, School of Life Sciences, Technical University of Munich, Emil-Ramann-Str. 4, 85354 Freising, Germany; liu.jinghui@tum.de; alexander.christmann@tum.de

[2]Chair of Crop Physiology, School of Life Sciences, Technical University of Munich, Alte Akademie 12, 85354 Freising, Germany; schaeufele@tum.de

[3]Chair of Root-Soil Interaction, School of Life Sciences, Technical University of Munich, Emil-Ramann-Str. 4, 85354 Freising, Germany; mutez.ahmed@tum.de

✉Correspondence: yang.zhenyu@tum.de

Article acceptance date: 3 July 2025

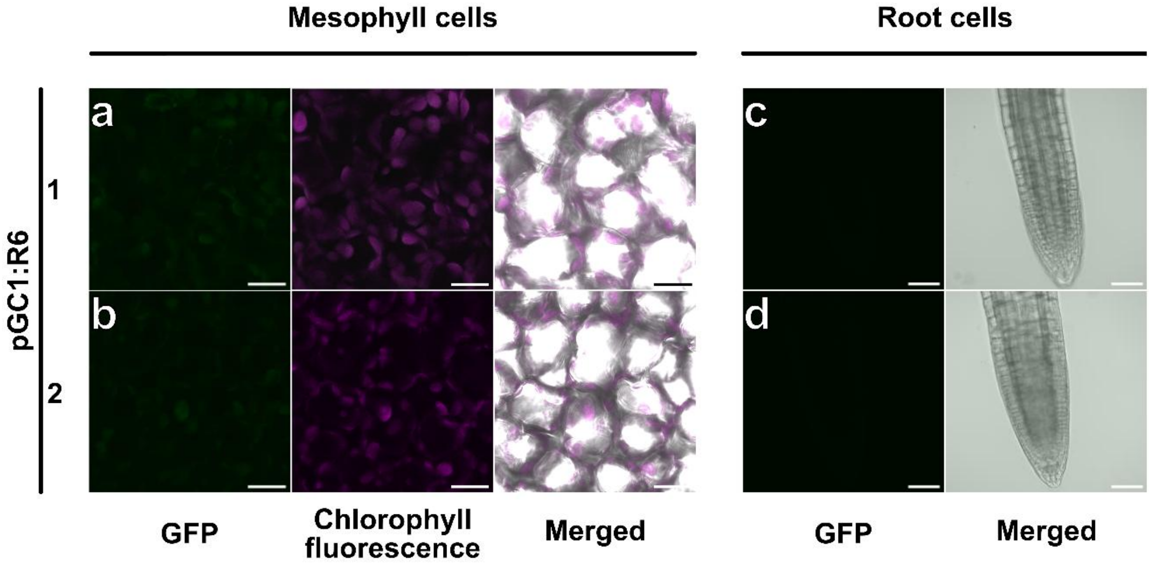

Fig. S1 The absence of GFP-R6 fusion protein in mesophyll and root cells. Representative confocal images of GFP (left panels), chlorophyll autofluorescence (middle panels; magenta), and composites of bright-field, GFP, chlorophyll autofluorescence images (right panels) in (a,b) palisade mesophyll cells. Representative confocal images of GFP (left panels) and composites of bright-field and GFP images (right panels) in (c,d) root cells. Palisade mesophyll cells of 14-day-old pGC1:R6 plants and root cells of 7-day-old pGC1:R6 seedlings were investigated. Scale bars in (a,b) and (c,d) represent 20 and 50 μm, respectively.

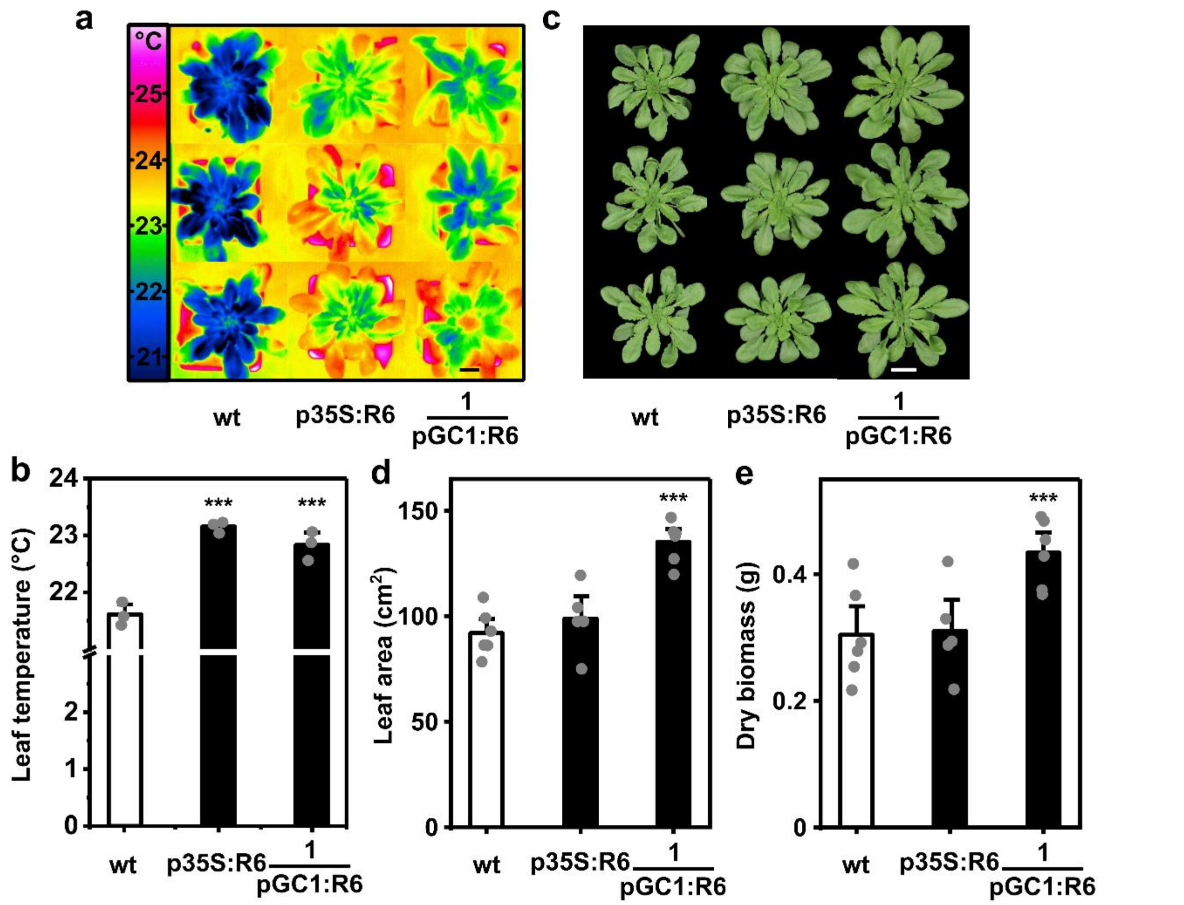

Fig. S2 Reduced leaf transpiration and sustained leaf growth in the pGC1:R6 line at 400 μmol m^{-2} s^{-1} PAR. (a) Thermal images and (b) leaf temperature values of 37-day-old wt, p35S:R6, pGC1:R6-1 line. (a,b) n = 3 biological replicates. (c) Representative rosette pictures, (d) projected leaf area, and (e) above-ground dry biomass of 36-day-old wt (n = 6), p35S:R6 (n = 5), pGC1:R6-1 line (n = 6 biological replicates). (a) The temperature was indicated as false color shown on the left panel. (a,c) Plants were randomized, and the images were aligned according to their genotype. The scale bars indicate 2 cm. (a to e) Mean ± SEM; ***$P < 0.001$ (one-way ANOVA) compared to wt.

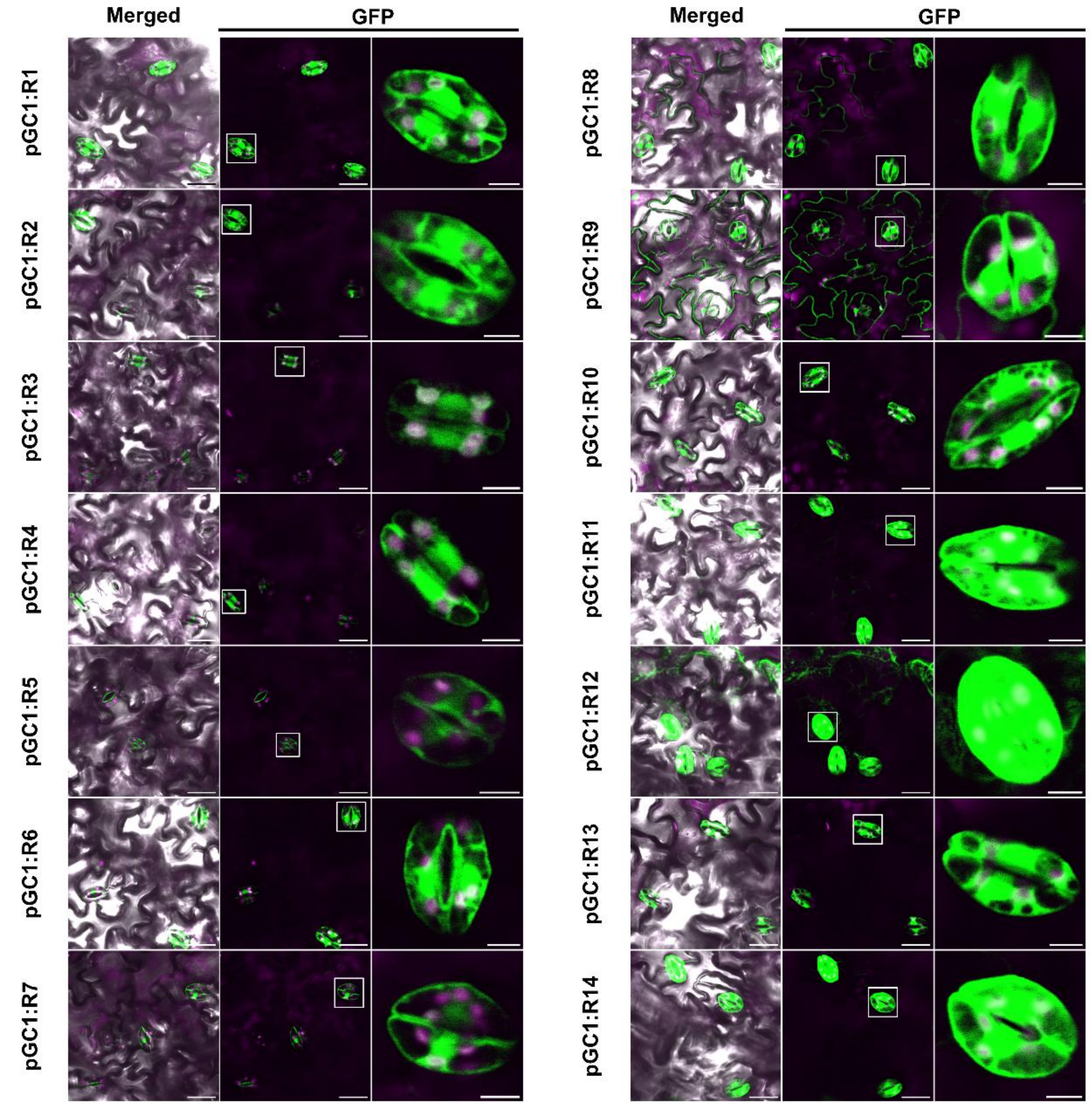

Fig. S3 Guard cell expression of ABA receptors driven by *pGC1* promoter. Representative confocal images showing fluorescent signals of pGC1:GFP-RCAR1 to RCAR14 (pGC1:R1 to14; one independent line shown for each receptor). The abaxial epidermis of 14-day-old plants was investigated. Left panels: bright-field images merged with GFP fluorescence (green) and chlorophyll autofluorescence (magenta). Middle panels: GFP fluorescence merged with chlorophyll autofluorescence. Right panels: magnified GFP fluorescence merged with chlorophyll autofluorescence of guard cells framed with white boxes shown on the middle panels. The scale bars on the left and middle panels indicate 20 μm, while the scale bars on the right panel indicate 5 μm.

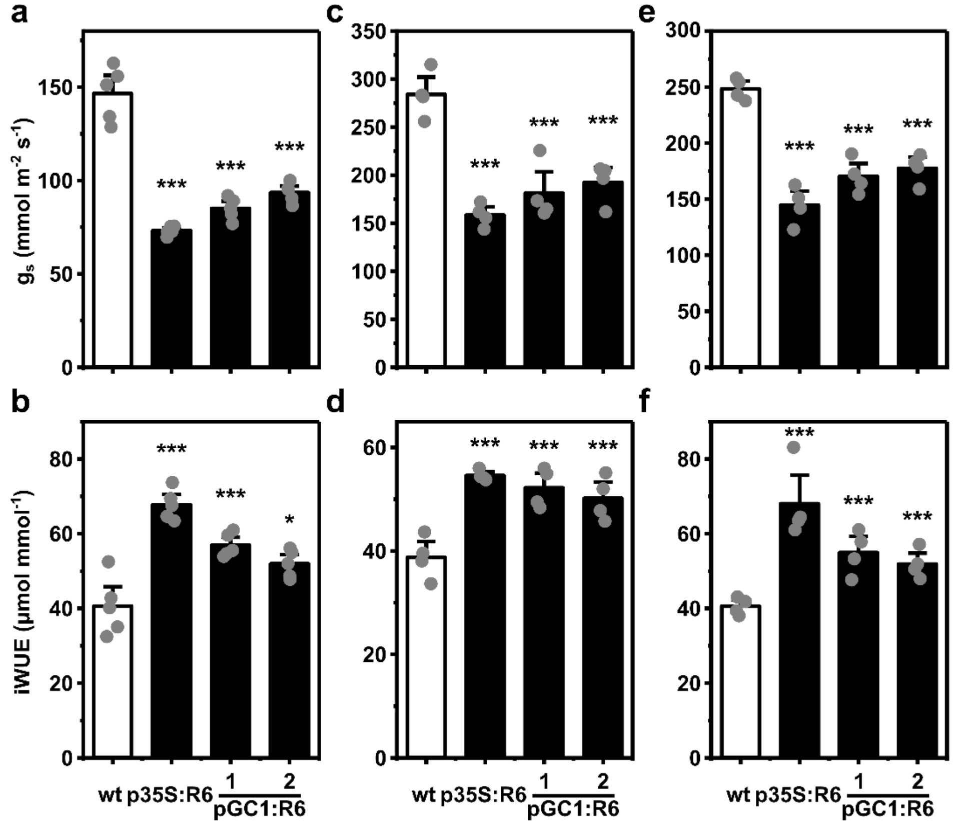

Fig. S4 Reduced stomatal conductance and enhanced intrinsic WUE in pGC1:R6 lines. Gas exchange analysis conducted (a,b) at 150 µmol m^{-2} s^{-1}, (c,d) 900 µmol m^{-2} s^{-1} PAR with whole-rosette configuration, and (e,f) 900 µmol m^{-2} s^{-1} PAR with single-leaf measurement. (a,c,e) Stomatal conductance (g_s) and (b,d,f) intrinsic WUE (iWUE) of wt (white columns), p35S:R6, and pGC1:R6 lines (back columns). (a,b) n = 5 and (c to f) n = 4 biological replicates, mean ± SEM; *P < 0.05 and ***P < 0.001 (one-way ANOVA) compared to the wt.

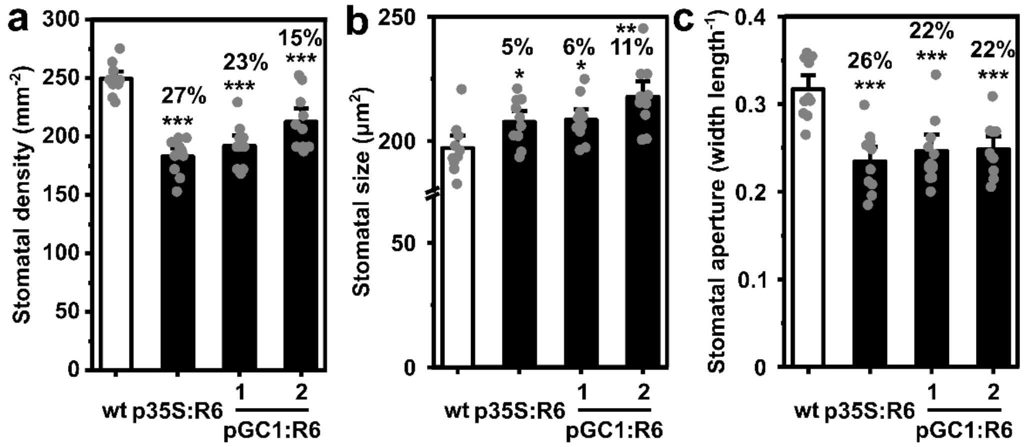

Fig. S5 Altered stomatal development and aperture in pGC1:R6 lines. (a) Stomatal density, (b) stomatal size, and (c) stomatal aperture of the 14th leaf of 25-day-old wild-type, p35S:RCAR6, and pGC1:RCAR6 plants. n = 10 biological replicates, mean ± SEM, *P<0.05, **P<0.01, and ***P<0.001 (one-way ANOVA) compared to wt.

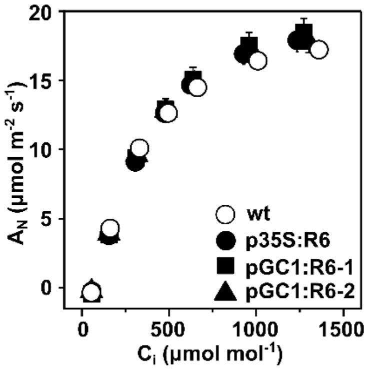

Fig. S6 The uncompromised A_N at varying intercellular CO_2 levels in pGC1:R6. A_N of Col (open circles), p35S:R6 (filled circles), pGC1:R6-1 (filled squares), and pGC1:R6-2 (filled triangles) in response to variable intercellular CO_2 levels (A-C_i curve). The gas exchange analysis was conducted using a whole-rosette configuration at 900 µmol m^{-2} s^{-1} PAR. ~~(A)~~ n = 8 biological replicates for the wt, n = 6 for p35S:R6, and n = 4 for pGC1:R6 lines, mean ± SEM.

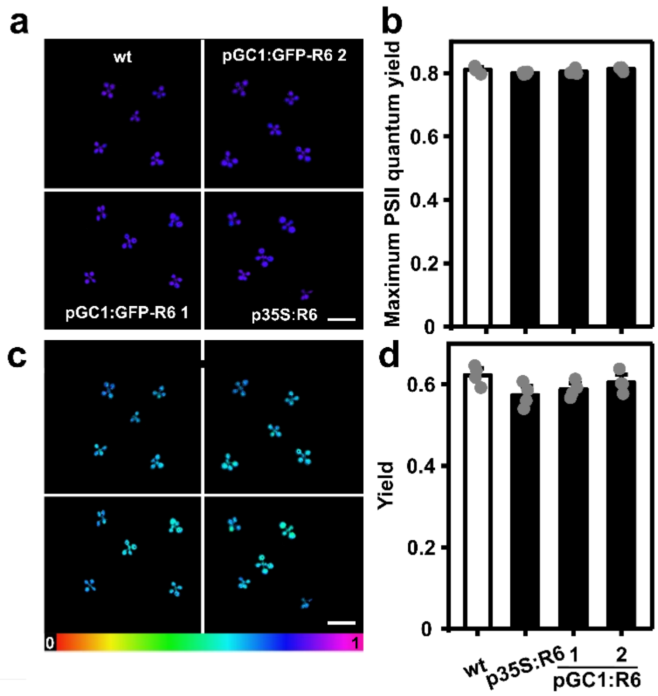

Fig. S7 Overexpressing RCAR6 in guard cells does not affect photosynthetic apparatus. (a) False-color images and (b) values of maximum photosystem II (PSII) quantum yield of 14-day-old wt (white column), p35S:R6, and pGC1:R6 lines (black columns). Maximum PSII quantum yield was determined using dark-adapted plants (dark adaption for 1 h). (c) False-color images and (d) values of PSII operating efficiency (yield) of the same plants in (a). Yield was determined 1 h after illumination (150µmol m^{-2} s^{-1} PAR). (a,c) Five individual plants of each genotype were grown in single pots under well-watered conditions. The images were recorded in a single experiment, and the layout was indicated in (a). The PSII efficiency is indicated as false colors as shown at the bottom of (c). The scale bars indicate 2 cm. (b,d) Mean ± SEM, and no significant difference was observed for p35S:R6 and pGC1:R6 lines compared to the wt.

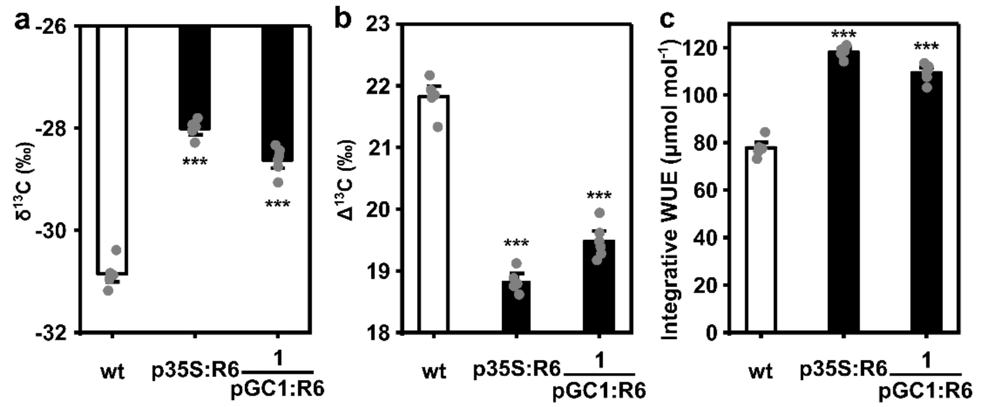

Fig. S8 Enhanced WUE of the pGC1:R6 line growth at 400 µmol m^{-2} s^{-1} PAR. (a) δ^{13}C, (b) Δ^{13}C, and (c) integrated WUE of leaf dry materials of wt (white columns), p35S:R6, and pGC1:R6 lines (back columns) in Fig. S2e. (a to c) n = 6 for wt, pGC1:R6-1, and n = 5 biological replicates for p35S:R6, mean ± SEM; ***P < 0.001 (one-way ANOVA) compared to wt.

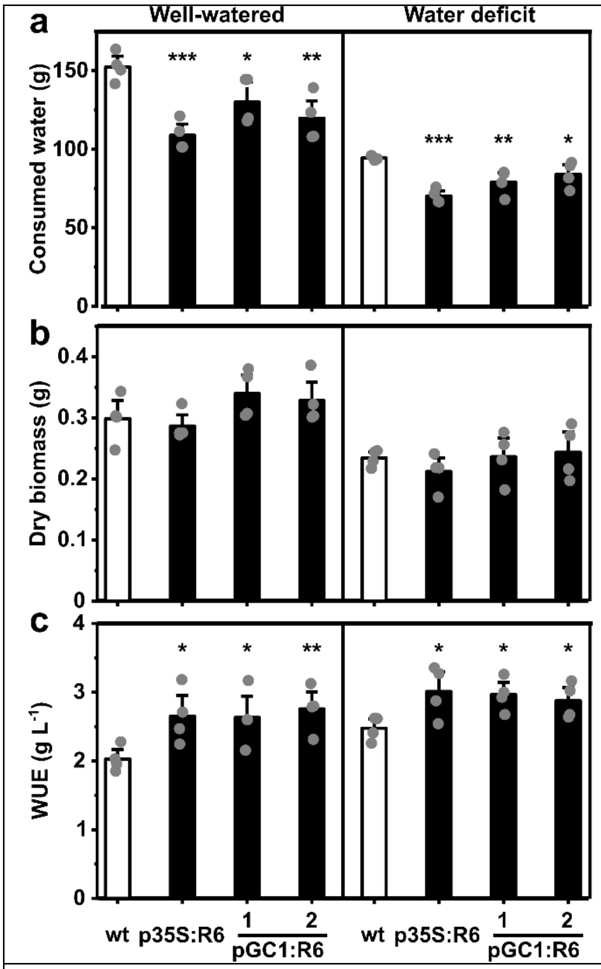

Fig. S9 Maintained biomass, reduced water consumption, and enhanced WUE of pGC1:RCAR6 lines under controlled water deficit conditions. (a) Consumed water, (b) above-ground dry biomass, and (c) WUE of 49-day-old wild-type, p35S:RCAR6, and pGC1:RCAR6 lines grown under well-watered conditions (around 60% soil water content) or controlled water deficit (around 20% soil water content). (a to c) n = 4 biological replicates, mean ± SEM; *P<0.05, **P<0.01, and ***P<0.001 (one-way ANOVA) compared to wt.

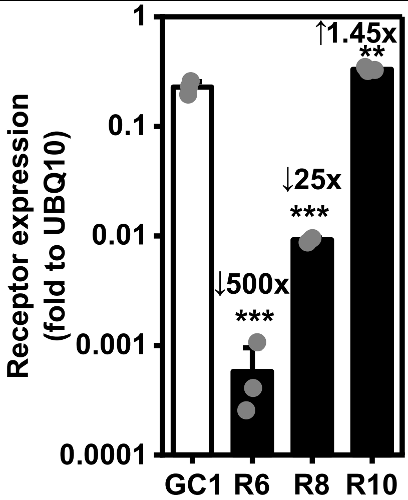

Fig. S10 Variation in transcript abundance of RCAR6 (R6), RCAR8 (R8), and RCAR10 (R10) in wild-type guard cells. Relative expression levels of each receptor were quantified by RT-qPCR and normalized to UBQ10 (black columns). GC1 transcript level (while column) serves as a guard cell-specific reference. Values above the black columns indicate fold change, and "↑" and "↓" indicate reduced or increased expression relative to GC1 expression. n = 3 biological replicates, mean ± SEM; **P<0.01, and ***P<0.001 (one-way ANOVA) compared to the expression of GC1.
